# Single Strand Annealing Plays a Major Role in RecA-Independent Recombination between Repeated Sequences in the Radioresistant *Deinococcus radiodurans* Bacterium

**DOI:** 10.1371/journal.pgen.1005636

**Published:** 2015-10-30

**Authors:** Solenne Ithurbide, Esma Bentchikou, Geneviève Coste, Bruno Bost, Pascale Servant, Suzanne Sommer

**Affiliations:** Institute for Integrative Biology of the Cell (I2BC), CEA, CNRS, Université Paris-Sud, Université Paris-Saclay, Gif sur Yvette, France; Brandeis University, UNITED STATES

## Abstract

The bacterium *Deinococcus radiodurans* is one of the most radioresistant organisms known. It is able to reconstruct a functional genome from hundreds of radiation-induced chromosomal fragments. Our work aims to highlight the genes involved in recombination between 438 bp direct repeats separated by intervening sequences of various lengths ranging from 1,479 bp to 10,500 bp to restore a functional *tetA* gene in the presence or absence of radiation-induced DNA double strand breaks. The frequency of spontaneous deletion events between the chromosomal direct repeats were the same in *recA*+ and in Δ*recA*, Δ*recF*, and Δ*recO* bacteria, whereas recombination between chromosomal and plasmid DNA was shown to be strictly dependent on the RecA and RecF proteins. The presence of mutations in one of the repeated sequence reduced, in a MutS-dependent manner, the frequency of the deletion events. The distance between the repeats did not influence the frequencies of deletion events in *recA* ^+^ as well in Δ*recA* bacteria. The absence of the UvrD protein stimulated the recombination between the direct repeats whereas the absence of the DdrB protein, previously shown to be involved in DNA double strand break repair through a single strand annealing (SSA) pathway, strongly reduces the frequency of RecA- (and RecO-) independent deletions events. The absence of the DdrB protein also increased the lethal sectoring of cells devoid of RecA or RecO protein. γ-irradiation of *recA* ^+^ cells increased about 10-fold the frequencies of the deletion events, but at a lesser extend in cells devoid of the DdrB protein. Altogether, our results suggest a major role of single strand annealing in DNA repeat deletion events in bacteria devoid of the RecA protein, and also in *recA* ^+^ bacteria exposed to ionizing radiation.

## Author Summary

*Deinococcus radiodurans* is known for its exceptional ability to tolerate exposure to DNA damaging agents and, in particular, to very high doses of ionizing radiation. This exceptional radioresistance results from many features including efficient DNA double strand break repair. Here, we examine genome stability in *D*. *radiodurans* before and after exposure to ionizing radiation. Rearrangements between repeated sequences are a major source of genome instability and can be deleterious to the organism. Thus, we measured the frequency of recombination between direct repeats separated by intervening sequences of various lengths in the presence or absence of radiation-induced DNA double strand breaks. Strikingly, we showed that the frequency of deletions was as high in strains devoid of the RecA, RecF or RecO proteins as in wild type bacteria, suggesting a very efficient RecA-independent process able to generate genome rearrangements. Our results suggest that single strand annealing may play a major role in genome instability in the absence of homologous recombination.

## Introduction

The extreme resistance of the bacterium *D*. *radiodurans* to DNA-fragmenting treatments, such as ionizing radiation or desiccation, is correlated with the ability to reconstruct a functional genome from hundreds of chromosomal fragments. The rapid reconstitution of an intact genome is thought to occur through an extended synthesis-dependent strand annealing process (ESDSA) followed by DNA recombination [1,2]. During ESDSA, chromosomal fragments with overlapping regions are used both as primers and templates for a massive synthesis of single-stranded DNA extensions. Newly synthesized complementary single stranded DNA extensions appear to anneal so that contiguous DNA fragments are joined together forming long linear intermediates. These intermediates require RecA-dependent homologous recombination to mature into reconstituted circular chromosomes representing DNA patchworks of numerous double-stranded DNA blocks synthesized before irradiation connected by DNA blocks synthesized after irradiation.

We have recently shown that the Deinococcal RecF, RecO, RecR proteins, by their ability to load RecA onto its single-stranded DNA substrate, play a crucial role in DNA double strand break repair via ESDSA and recombinational repair pathways [3]. Mutant Δ*uvrD* bacteria showed a markedly decreased radioresistance, an increased latent period in the kinetics of DNA double strand break repair and a slow rate of fragment assembly correlated with a slow rate of DNA synthesis, suggesting that UvrD helicase might be involved in the processing of double stranded DNA ends and/or in the DNA synthesis step of ESDSA [3].

More recently, it was proposed that a single strand annealing (SSA) process participates in an early step of DNA double strand break repair by facilitating the accurate assembly of small fragments to generate suitable substrates for subsequent ESDSA-promoted genome reconstitution [4]. The DdrB protein was previously shown to exhibit *in vitro* properties akin to those of SSB protein [5] but also to promote annealing of single stranded DNA [6]. The DdrB protein, recruited early after irradiation into the nucleoid, was also shown to be involved in the slow DNA double strand break repair observed in cells devoid of the RecA protein, and thus to play a major role in RecA-independent DNA double strand break repair through SSA [4,6].

Rearrangements between repeated sequences are a major source of genome instability and can be deleterious to the organism. These rearrangements can result in deletion or duplication of genetic material flanked by direct repeats. In *Escherichia coli*, recombination between directly repeated sequences occurs via both RecA-independent and RecA-dependent mechanisms, depending on the size of the repeats and of the intervening sequences between the repeated sequences [7–9]. Insertion of a sizable DNA sequence in between the repeated sequences substantially increased the RecA dependence, suggesting that increasing the distance separating the homologous regions preferentially inhibits the RecA-independent recombination in *E*. *coli* [9,10]. In *E*. *coli*, RecA-independent rearrangements between short repeats, such as deletions, are stimulated by mutations that affect the DNA polymerase or other proteins involved in DNA replication [11–13] leading to the proposal that these events occur during DNA replication by a mechanism involving mispairing of the newly synthetized DNA strand with an alternative complementary template sequence located nearby [7,12] (for review, see [14,15]). An alternate mechanism for RecA-independent deletion events involves DNA breakage, exonucleolytic erosion of the DNA ends and single strand annealing (SSA) of exposed complementary single stranded DNA [14]. A single strand annealing mechanism has also been proposed for RecA-independent deletions associated with a restart of collapsed replication forks [7,11,12,14,16]. Here, we measured the frequency of recombination between direct repeats separated by intervening sequences of various lengths restoring a functional *tetA* gene in the presence or absence of radiation-induced DNA double strand breaks in *D*. *radiodurans*. We also assessed the involvement of the RecA, RecO, RecF, UvrD and DdrB proteins in the deletion process. The role of these proteins in the progression of replication forks was also discussed.

## Results

### Efficient RecA-independent recombination between direct repeats in *D*. *radiodurans*

**Fig 1:**
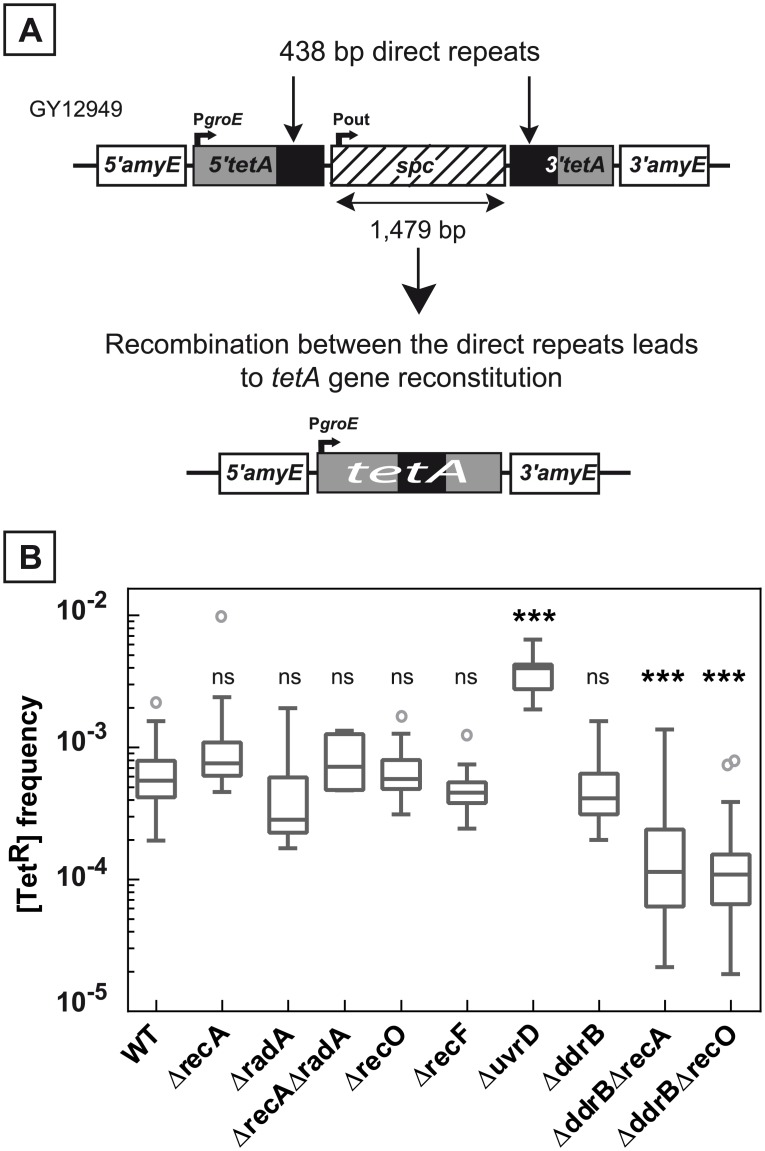
Deletion frequency between repeated sequences depends on the genetic context. **A.** Schematic representation of the genetic recombination assay. The 5’ and 3’ parts of the *tetA* gene, containing 438 bp repeats (black boxes) separated by an intervening sequence of 1,479 bp encoding a *spc* gene conferring resistance to spectinomycin, were introduced into the dispensable *amyE* gene locus. Recombination between the repeats leads to the *tetA* gene reconstitution conferring tetracycline resistance to bacteria. **B.** Medians of [Tet^R^] frequencies in WT (GY12949), Δ*recA* (GY15184), Δ*radA* (GY12956), Δ*recA*Δ*radA* (GY12971), Δ*recO* (GY12963), Δ*recF* (GY12955), Δ*uvrD* (GY12953), Δ*ddrB* (GY16016), Δ*ddrB* Δ*recA* (GY16628), and Δ*ddrB* Δ*recO* (GY16638) mutant strains are represented by Tukey box plots. Outliers are represented by open circles. Medians were calculated from 12 to 53 independent values (except for medians calculated from at least 4 independent values in Δ*radA* and Δ*recA* Δ*radA* bacteria). Statistically significant differences in the medians of recombination frequencies of the mutants, compared to those observed in strain GY12949, were calculated using the non-parametric Dunn's multiple comparison test: * P < 0.05; ** P < 0.01; *** P < 0.001; ns if P > 0.05.

To investigate the role of recombination proteins in the occurrence of repeat-mediated deletion events in *D*. *radiodurans*, we constructed a mutated *tetA* allele bearing an internal duplication and a *spc* cassette inserted between the duplicated regions. The engineered *tetA* allele was inserted into the dispensable *amyE* locus of chromosome 1 and provided a recombination substate in which the direct repeats (438 bp long) were separated by a 1,479 bp spacer (Fig 1A). Precise deletion of one of the direct repeats and the intervening sequence restores the wild type *tetA* allele. The presence of a functional *tetA* gene on one copy of chromosome 1 suffices to confer tetracycline resistance to the cells, although *D*. *radiodurans* bacteria contain 4 to 10 genome equivalents per cell. In contrast, when the deletion of a gene generates a loss-of-function mutant, all the copies of the gene must be eliminated to detect the mutant phenotype, and failure to obtain a homozygote provides a first indication that the gene might encode a function essential for cell viability [3,17].

The frequency of spontaneous deletion events in a population was estimated by measuring the frequency of [Tet^R^] bacteria. As shown in Fig 1B, the median of the frequencies of [Tet^R^] in the wild type bacteria was 6.5x10^-4^ and did not decrease in Δ*recA* bacteria (median value: 8x10^-4^). Does the apparent RecA-independent high frequency of deletion events result from a functional redondancy of RecA activities in the cells? We tested the involvement of the RadA protein, a RecA-related protein, and showed that wild type frequencies of deletions were found in Δ*radA* and in Δ*recA* Δ*radA* bacteria (Fig 1B), suggesting that the RadA protein was not required in RecA-independent recombination to compensate for the absence of the RecA protein. We also observed that the frequency of [Tet^R^] bacteria was not reduced in cells devoid of RecF or RecO proteins required for loading RecA onto its single-stranded DNA substrate (Fig 1B). Altogether, our results suggest an important contribution of RecA-independent mechanisms in the generation of deletions between repeated DNA sequences in *D*. *radiodurans*.

**Fig 2:**
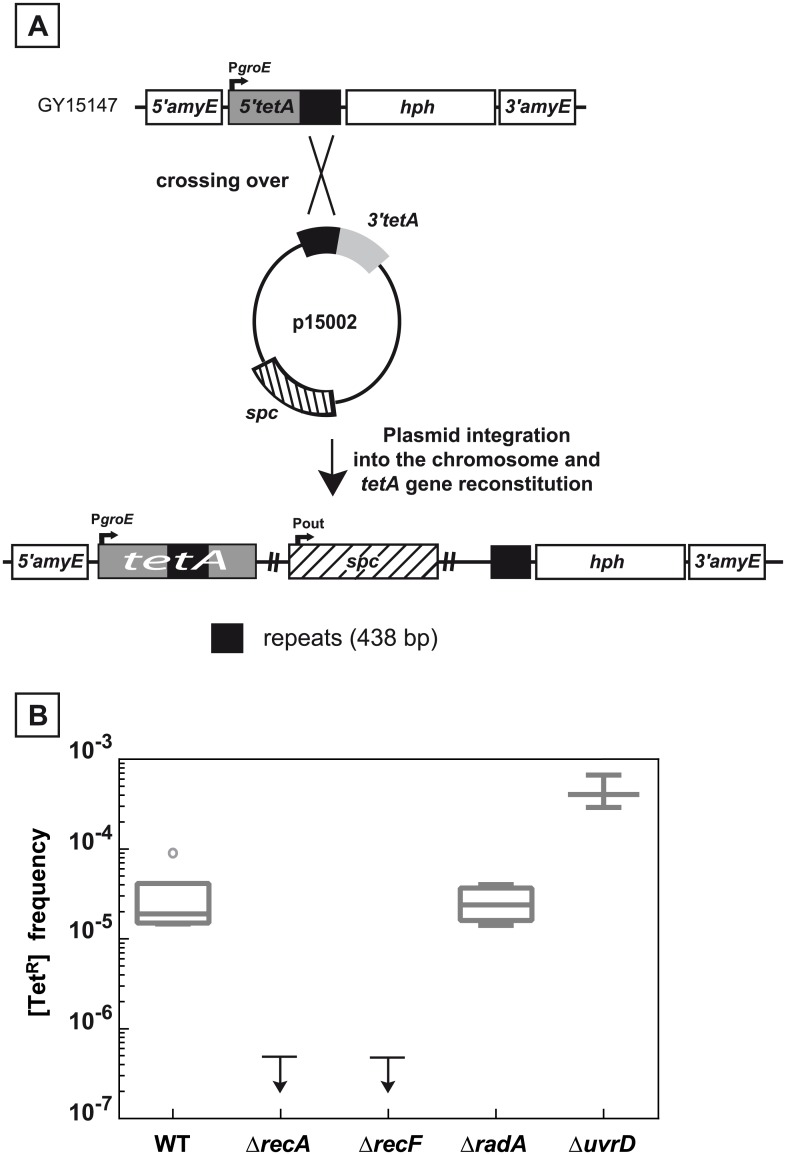
Recombination between chromosomal and plasmid DNA is RecA- and RecF-dependent. **A.** Schematic representation of the recombination assay between chromosomal and plasmid DNA. The 5’*tetA* and the 3’*tetA* regions of the *tetA* gene, containing 438 bp repeats, were introduced into the chromosomal dispensable *amyE* gene and into the p11554 plasmid giving rise to plasmid p15002, respectively. One crossing over between the two 438 bp repeated sequences (black boxes) leads to the reconstitution of a functional *tetA* gene and the integration of the plasmid into chromosomal DNA. **B.** Medians of [Tet^R^] frequencies in WT (GY15147), Δ*recA* (GY15158), Δ*recF* (GY15160), Δ*radA* (GY15149), and Δ*uvrD* (GY15156) bacteria, all containing the p15002 plasmid, are calculated from at least 3 independent values and represented by Tukey boxplots. Outliers are represented by open circles. The small arrows attached to the horizontal line representing the upper limit of detectable [Tet^R^] frequencies indicate that [Tet^R^] frequencies were < 5 10^−7^ for Δ*recA* and Δ*recF* bacteria.

To test whether the same 438 bp homologous fragments can undergo efficient RecA-dependent recombination, we constructed a plasmid-by-chromosome recombination assay in which the recombining *tetA* fragments were placed in a different configuration, one being located at the chromosomal *amyE* locus and the other on a resident plasmid (Fig 2A). In this assay, the reconstitution of a functional *tetA* gene resulted from the integration of the plasmid into the chromosomal DNA as verified by PCR analysis of few [Tet^R^] colonies (S1 Fig). The integration event is well tolerated by the cell, since we used a low copy number plasmid p15002, a derivative of plasmid pI8 maintained at 4 to 10 copies per cell in *D*. *radiodurans* [18]. As can be seen in Fig 2B, the frequency of [Tet^R^] bacteria dropped from a median value of 2x10^-5^ in the wild type to less than 5 x 10^−7^ in cells devoid of the RecA or the RecF proteins, indicating that the 438 bp homologous fragments recombine through a classical RecA-promoted strand exchange mechanism. In contrast, loss of the RadA protein did not impair recombination efficiency. In *E*. *coli*, the RadA protein has been involved in processing of branched recombination intermediates. However single *radA* mutants have a modest effect on recombination and DNA survival while they show a strong synergistic effect in combination with mutations in the *recG* or the *ruvAB* Holliday junction proteins [19,20].

### Differences between the DNA repeats reduces the frequency of the deletion events

**Fig 3:**
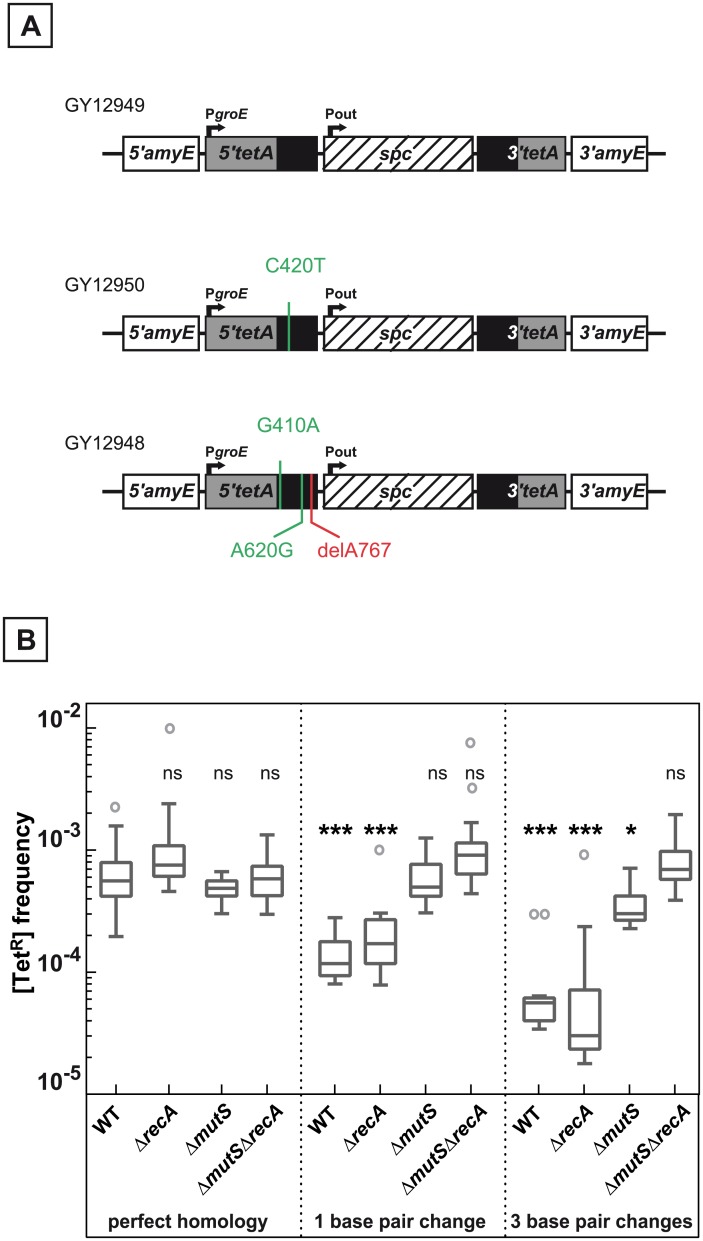
Effect of base pair changes in the repeats on their recombination frequencies. **A.** Schematic representation of the recombination substrates containing 1 or 3 bp changes. The positions of the base pair changes calculated from the initiation codon in the 5’*tetA* region are indicated. **B.** Medians of [Tet^R^] frequencies calculated from 14 to 43 independent values in WT (GY12949), Δ*recA* (GY15184), Δ*mutS* (GY12978), Δ*mutS ΔrecA* (GY16620) bacteria containing identical repeated sequences, WT (GY12950), Δ*recA* (GY16624), Δ*mutS* (GY12980), Δ*mutS* Δ*recA* (GY16618) containing one base difference in the repeated sequences, and WT (GY12948), Δ*recA* (GY16622), Δ*mutS* (GY12979), Δ*mutS* Δ*recA* (GY16616) containing 3 base differences in the repeated sequences, are represented by Tukey boxplots. Outliers are represented by open circles. Statistically significant differences in the medians of recombination frequencies of the mutants and WT containing sequence differences in the repeated sequence compared to GY12949 were calculated using the non-parametric Dunn's multiple comparison test: * P < 0.05; ** P < 0.01; *** P < 0.001; ns if P > 0.05.

The presence of mutations in one of the 438 bp repeats (Fig 3A) reduced the frequency of the deletion events between the chromosomal repeats. A single mutation sufficed to significantly decrease the frequency of the deletion events as shown by median values that decreased by a factor of 4.8 in *recA* ^+^ bacteria and 4.6 in Δ*recA* bacteria, when compared to fully homologous repeats. The decrease was greater when 3 mutations were present in one of the repeats, yielding reduction factors of 10.0 and 19.0 in *recA* ^+^ and Δ*recA* bacteria, respectively (Fig 3B). A plot of the frequency of [Tet^R^] bacteria as a function of the number of differences shows a linear decrease in the deletion frequency with similar regression slopes in *recA* ^+^ and Δ*recA* bacteria (S2 Fig).

A similar analysis was performed in *recA* ^+^ Δ*mutS* and Δ*recA* Δ*mutS* bacteria devoid of the MutS protein, the key enzyme involved in mismatch recognition. The data (Fig 3B and S2 Fig) show that in this case the differences between the repeats did not significantly affect the deletion frequency. These results suggest that, as in homologous recombination intermediates, a heteroduplex DNA is formed during RecA-independent processes leading to the reconstitution of a functional *tet* gene and that an efficient mismatch repair aborts recombination between the DNA repeats in Δ*recA* as well as in *recA* ^+^ bacteria.

### Absence of UvrD increases the frequency of deletions between the repeats

It was previously shown that *uvrD* mutations stimulate RecA-dependent recombination [21–23] and enhance tandem repeat deletions in *E*. *coli* [23]. Here, we show that the absence of UvrD enhanced the efficiency of RecA-dependent recombination between chromosomal and plasmid DNA by a factor of 21.2 (Fig 2B), suggesting that deinococcal UvrD protein possesses an anti-RecA activity as previously shown for the *E*. *coli* UvrD protein [24,25]. The absence of UvrD also enhanced the frequency of deletions between the chromosomal direct repeats by a factor of 7.1 (Fig 1B). This increase may be due to the anti-RecA activity of UvrD protein that can possibly inhibit RecA-dependent recombination between the repeated sequences in a *recA* ^+^ *uvrD* ^+^ background. However, the absence of UvrD might also disturb DNA replication, and thus increase genome instability. A clue to understand how the absence of the UvrD protein might be involved, independently of its anti-RecA activity, in the stimulation of deletion events requires an analysis of its effects in a recombination-deficient background. Unfortunately, we were unable to obtain homozygotes for *recA*, *recF* or *recO* deletion in combination with a *uvrD* deletion, even after extensive purification steps (S3 Fig), suggesting that *uvrD* deletion is colethal with a *recA*, *recF* or *recO* deletion. We propose that the UvrD protein, by displacing obstacles downstream of the replisome, plays an important role in the progression of replication forks (see discussion).

### Loss of viability of Δ*ddrB* Δ*recA* and Δ*ddrB* Δ*recO* double mutants

**Fig 4:**
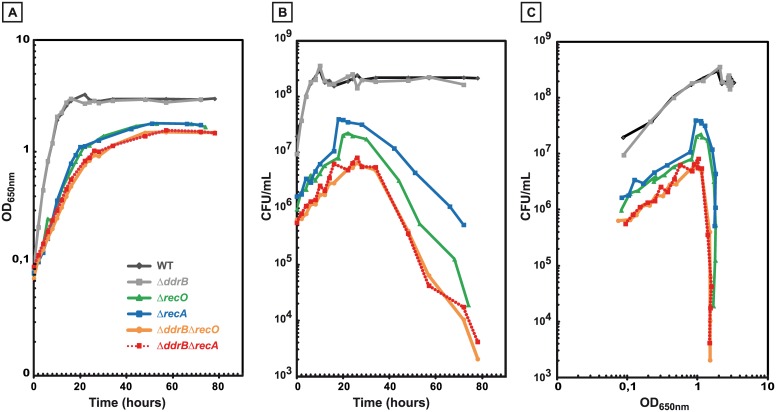
Impaired growth and stationary-phase lethality of recombination-deficient mutant cells. GY9613 (WT) (black diamonds), GY13915 (Δ*ddrB*) (grey squares), GY15125 (Δ*recO*) (green triangles), GY12968 (Δ*recA*) (blue squares), GY16626 (Δ*ddrB* Δ*recA*) (red squares and interrupted lines), GY16636 (Δ*ddrB* Δ*recO*) (orange circles) were grown from independent colonies at 30°C to an OD_650nm_ = 0.1 (time 0 of the growth curves). **A.** OD_650nm_ as a function of time. **B.** Colony forming units as a function of time. **C.** Colony forming units as a function of OD_650nm_.

Previous *in vitro* and *in vivo* results suggest that the DdrB protein plays a major role in a single strand annealing process (SSA) that operates early in genome reconstitution after DNA damage [4,6]. Single strand annealing is the only activity of DdrB known besides binding to single strand DNA [5,6]. Thus, to analyse the involvement of SSA in generating deletions via a RecA-independent pathway, we decided to construct double mutants devoid of DdrB and RecA or RecO proteins. Homogenotization of Δ*ddrB* Δ*recA* and Δ*ddrB* Δ*recO* double mutants was difficult, requiring 7 steps of purification, suggesting growth inhibition of the mutated cells (S3 Fig). Thus, we compared the growth rate and the plating efficiency of the double mutants with those of the single Δ*ddrB*, Δ*recA* and Δ*recO* mutants and of the parental wild type strain. Wild type and Δ*ddrB* bacteria exhibited a generation time of 105 min. The recombination deficient bacteria grew more slowly, as Δ*recA* and Δ*recO* bacteria during exponential growth showed a generation time of 285 min whereas Δ*ddrB* Δ*recO* and Δ*ddrB* Δ*recA* exhibited a generation time of 370 min. Moreover, during exponential growth phase, the single Δ*recA* and Δ*recO* mutants had a 15 fold decreased plating efficiency as compared with the wild type, whereas the Δ*ddrB* Δ*recA* and Δ*ddrB* Δ*recO* double mutants had a 35 fold decreased plating efficiency (Fig 4). These results suggest that the DdrB protein may be involved in management of blocked replication forks in the absence of the RecA or RecO proteins. Another striking result was the increased lethality of the recombination deficient mutants in late stationary phase. Indeed, after reaching a plateau after 6 hours of incubation for the wild type and Δ*ddrB* bacteria, the number of CFU did not decrease during 70 additional hours of incubation. In contrast, the single Δ*recA* and Δ*recO* recombination deficient mutants and the double Δ*ddrB* Δ*recA* and Δ*ddrB* Δ*recO* mutants reached a plateau after 18 to 20 hours of incubation and the number of CFU decreased 2–3 orders of magnitude after 30 hours of incubation (Fig 4) suggesting that DNA lesions are generated during prolonged stationary phase and require recombination functions to be repaired.

### An important role of DdrB in the recombination process between repeated sequences in the absence of RecA or RecO proteins

We found that the absence of DdrB had a strong negative effect on the frequencies of deletions events between the chromosomal repeats generated via a RecA-independent pathway. Indeed, when the repeats are separated by 1,479 bp, the median values of the deletion frequencies in Δ*ddrB* Δ*recA* and Δ*ddrB* Δ*recO* bacteria decreased by a factor of 4.9 and 5.1 respectively, as compared to their Δ*recA* and Δ*recO* counterparts (Fig 1B). These results indicate that 80% of the [Tet^R^] bacteria generated in the absence of RecA or RecO proteins were formed in a DdrB-dependent manner, suggesting a major role of single strand annealing in RecA-independent recombination between the direct repeats. We can also hypothesize that the DdrB protein might be involved in the stabilization of DNA polymerase template switching intermediates.

### The frequency of deletion events does not decrease when the length of the spacers between the repeated sequences was as large as 10,500 bp

**Fig 5:**
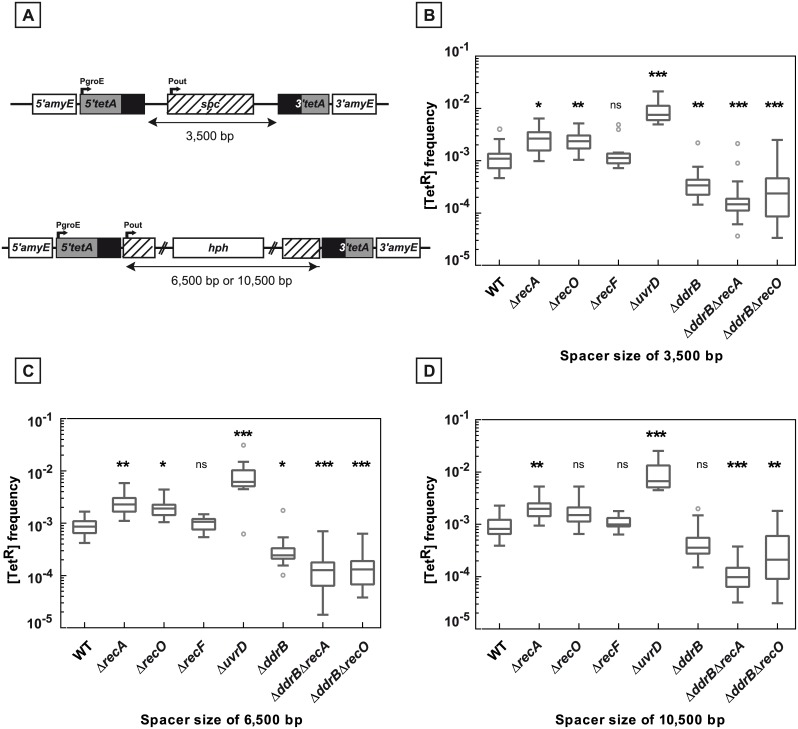
Deletion frequencies between repeated sequences separated by 3,500, 6,500, and 10,500 bp. **A.** Schematic representation of the constructions used. **B., C., D.** Bacteria contain 3,500 bp (panel B), 6,500 bp (panel C), and 10,500 bp (panel D) intervening sequences between the *tetA* repeats. The medians of [Tet^R^] frequencies calculated from 10 to 35 independent values in the tested strains are represented by Tukey boxplots. Outliers are represented by open circles. Statistically significant differences in the medians of recombination frequencies of the mutants compared to the WT GY16209, GY16227, and GY16235 in panel B, C, and D, respectively, were calculated using the non-parametric Dunn's multiple comparison test: * P < 0.05; ** P < 0.01; *** P < 0.001; (ns) if P > 0.05. **B.** WT (GY16209), Δ*recA* (GY16238), Δ*recO* (GY16262), Δ*recF* (GY16264), Δ*uvrD* (GY16608), Δ*ddrB* (GY16268), Δ*ddrB* Δ*recA* (GY16630), Δ*ddrB* Δ*recO* (GY16640) **C.** WT (GY16227), Δ*recA* (GY16244), Δ*recO* (GY16278), Δ*recF* (GY16276), Δ*uvrD* (GY16612), Δ*ddrB* (GY16282), Δ*ddrB* Δ*recA* (GY16632), Δ*ddrB* Δ*recO* (GY16642) **D.** WT (GY16235), Δ*recA* (GY16252), Δ*recO* (GY16290), Δ*recF* (GY16292), Δ*uvrD* (GY16614), Δ*ddrB* (GY16296), Δ*ddrB ΔrecA* (GY16634), Δ*ddrB ΔrecO* (GY16644).

In *E*. *coli*, in which both RecA-dependent and RecA-independent mechanisms can contribute to recombination between direct repeats, deletion events become increasingly RecA-dependent as the distance between the repeated sequences increases [9]. To verify if this also applies in *D*. *radiodurans*, we modified the test deletion construct shown in Fig 1 by replacing the 1,479 bp spacer with sequences of increasing length to analyse the impact of the distance between the repeats on the incidence of deletions and their genetic control (Fig 5A).

We found that the increase of the distance between the repeats from 1,479 bp (Fig 1A) up to 10,500 bp (Fig 5A) had no effect on the deletion frequency in *recA* ^+^ as well as in Δ*recA* or Δ*recF* hosts (compare the [Tet^R^] frequencies in Figs 1B, 5B, 5C and 5D). Likewise, the distance between the repeats had no effect on the stimulation of deletion events by the absence of the UvrD protein (Figs 1B, 5B, 5C and 5D).

In contrast, the involvement of DdrB in the deletion events became more apparent when the distance between the repeats increased. Indeed, while a DdrB deficiency had no effect on the frequency of deletions in a *recA* ^+^ background when the spacer between the repeats was 1,479 bp long, it produced a 2 to 3-fold decrease in the deletion frequency when the spacer length increased (Figs 1B, 5B, 5C and 5D). When the *ddrB* deletion was associated with a *recA* deletion, the reduction factors were found to be between 18- and 20-fold if the length of the intervening sequences was ≥ 3,500bp as compared to their single Δ*recA* counterparts (Fig 5B, 5C and 5D). A similar effect of a *ddrB* deletion was also observed in cells devoid of the RecO protein (Fig 5B, 5C and 5D). These results suggest that, in the absence of the RecA-promoted homologous recombination, approximately 95% of the recombination events were dependent on the DdrB protein and may be related to an SSA pathway.

### Induction of recombination between direct repeats by ionizing radiation

**Fig 6:**
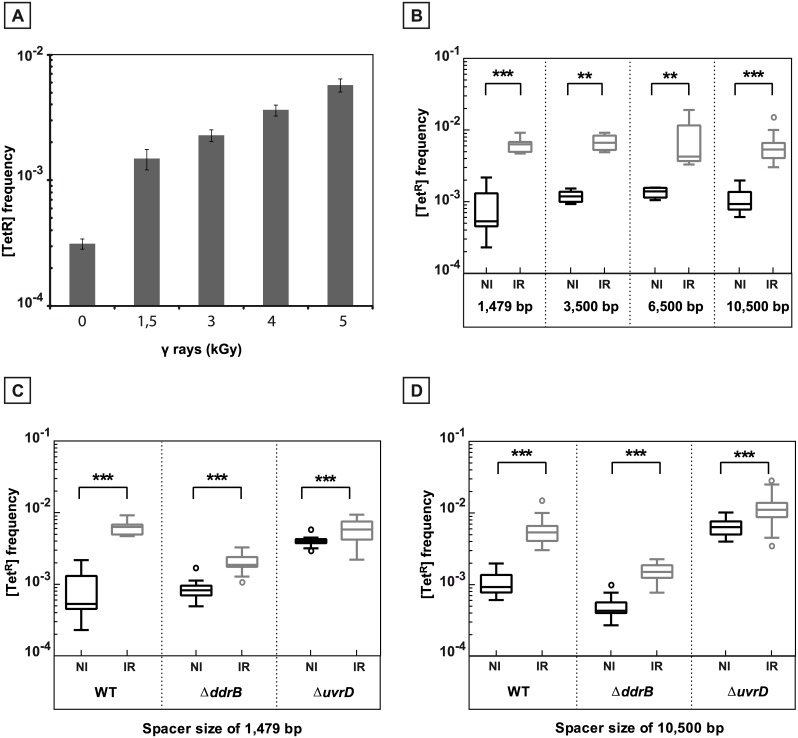
γ-irradiation induced recombination between repeated sequences. **A.** Induction of recombination between repeated sequences separated by 1,479 bp as a function of the radiation dose. [Tet^R^] frequencies were measured in at least 5 independent cultures after 20 hours of post irradiation incubation of GY12949 in TGY medium after exposure to different γ-irradiation doses. **B.** Induction by exposure to γ-irradiation of recombination between repeats separated by intervening sequences of increasing length: 1,479 bp (GY12949), 3,500 bp (GY16209), 6,500 bp (GY16227) and 10,500 bp (GY16235). **C.** γ-promoted induction of recombination between overlapping sequences separated by 1,479 bp in WT (GY12949), Δ*ddrB* (GY16016), and Δ*uvrD* (GY12953) bacteria. **D.** γ-promoted induction of recombination between overlapping sequences separated by 10,500 bp in WT (GY16235), Δ*ddrB*(GY16296), Δ*uvrD*(GY16614). **B.**, **C.**, **D.** Medians of the [Tet^R^] frequencies calculated from 5 to 30 independent values are represented by Tukey boxplots. Outliers were represented by open circles. Statistically significant differences in the medians of recombination frequencies between irradiated and the corresponding non-irradiated bacteria were calculated using the non-parametric Mann-Withney test: * P < 0.05; ** P < 0.01; *** P < 0.001; (ns) if P> 0.05. NI: non-irradiated bacteria. IR: irradiated bacteria.

We used our deletion assay to analyze the impact of the presence of repeated sequence on the stability of the genome in γ-irradiated cells during the process of genome reconstitution. We showed that the frequency of repeat-induced deletions restoring a functional *tetA* gene increased as a function of the dose of γ-irradiation used (Fig 6A). Thus, we further exposed the cells to 5 kGy γ-irradiation, a dose producing hundreds of DNA double strand breaks [26]. The repeats in the tested cells were separated either by 1,479 bp (“short” spacer), 3,500 bp, 6,500 bp, or by 10,500 bp (“long” spacer) sequences. The deletion analysis was performed only in a *recA* ^+^ background, since the extreme radio-sensitivity of Δ*recA* or Δ*recF* bacteria (cell survival was less than 10^−5^ after exposure to 5 kGy) precluded the inclusion of these cells in the genetic assay. The deletions were induced by exposure to γ-irradiation independently of the length of the spacers (Fig 6B).

As shown in Fig 6, in the wild type bacteria, the deletion frequency increased by 11.9 fold and 5.9 fold after irradiation when the repeats are separated by the”short” spacer and the “long” spacer, respectively (compare left panels in Fig 6C and 6D). In cells devoid of the UvrD protein, the frequency of deletions moderately increased after irradiation (induction factors of 1.45 and 1.7 for the “short” and “long” spacer, respectively), likely because these cells already have an elevated spontaneous level of recombination in the absence of irradiation (compare right panels in Fig 6C and 6D). In contrast, cells devoid of the DdrB protein showed a marked reduction in the induced levels of deletion events (compare middle panels in Fig 6C and 6D), suggesting that single strand annealing might play an important role in the generation of the [Tet^R^] recombinants during genome reconstitution after irradiation.

## Discussion

Repeated sequences are targeted by recombination processes leading to amplifications, deletions, and other rearrangements of the genetic material. These events play an important role in genome plasticity and rapid adaptation to environmental challenges, but are also potential source of genome instability and can be deleterious to an organism (see for review [27]). *D*. *radiodurans* contains an enhanced number of repetitive sequences as compared to other bacteria, including insertion sequences, small non-coding repeats, and intragenic repeats (see for review [28]). This bacterium is also known for its capacity to reconstitute an entire genome from a myriad of fragments after exposure to elevated γ-irradiation doses. Genome reconstitution occurs through extended synthesis dependent single strand annealing (ESDSA) followed by classical homologous recombination. In heavily irradiated cells, a RecA-independent single strand annealing (SSA) process takes place before ESDSA allowing the assembly of small fragments into substrates that can be further processed through ESDSA. The single strand annealing activity of the DdrB protein plays a major role in this early step of DNA double strand break repair [4].

Here, we show that DdrB also plays a key role in RecA-independent recombination between direct repeats, leading to the occurrence of deletions, and substantially contributes to the induction of deletions in γ-irradiated *recA* ^+^ bacteria. Our results points to an important contribution of a DdrB-dependent pathway in *D*. *radiodurans* genome plasticity.

### Efficient mechanisms ensure recombination between chromosomal direct repeats in *D*. *radiodurans* cells devoid of the RecA protein

Here, we determined the frequencies of spontaneous and radiation-induced recombination between chromosomal direct repeats and investigated the role of RecA and of other key recombination and repair proteins in the occurrence of these events in *D*. *radiodurans*. We found that recombination events restoring a functional *tetA* gene occurred at a very high frequency. In a *recA* ^+^ background, the median frequency of [Tet^R^] cells was equal to 6.5 x 10^−4^ (Figs 1, 5B, 5C and 5D), values more than 10-fold higher than those measured in a study that used a similar substrate (chromosomal 358 bp direct repeats separated by an intervening sequence of 850 bp) to measure recombination in *Helicobacter pylori*, a bacterium known for its high recombination proficiency [29]. Moreover, inactivation of *recA* resulted in a 10-fold decrease in recombination between the direct repeats in *H*. *pylori* [29]. In contrast, introduction of a Δ*recA*, Δ*recF* or Δ*recO* mutation in the *D*. *radiodurans* tester strains did not change the elevated recombination frequencies between the repeats (Figs 1, 5B, 5C and 5D). In *E*. *coli* and in *B*. *subtilis*, the distance between the repeats plays a key role in determining the mechanisms involved in the recombination processes, the efficiency of RecA-independent recombination decreasing sharply when the distance between the repeats increases [9, 10, 30]. No such proximity effect was observed in *D*. *radiodurans*. Indeed, the frequency of appearance of the recombinants in our assay remained elevated in Δ*recA* (and Δ*recF* or Δ*recO*) bacteria as in the parental *rec* ^+^ bacteria when the spacer between the repeats increased from 1,479 up to 10,500 bp (Fig 5).

Our results suggest that, in the absence of RecA (or in the absence of “facilitator” proteins required for loading RecA onto its single-stranded DNA substrate), alternate pathways ensure recombination between repeated sequences in *D*. *radiodurans*. These RecA-independent pathways do not necessarily predominate in *recA* ^+^ bacteria, although we observed a similar frequency of recombinants in *recA* ^+^ and Δ*recA* bacteria. Indeed, Δ*recA* (or Δ*recF* or Δ*recO*) bacteria had a low plating efficiency with less than 10% of cells able to form colonies.

### Does replication slippage account for RecA-independent recombination between chromosomal direct repeats?

In *E*. *coli*, mutations in components of the DNA Pol III holoenzyme result in elevated levels of tandem repeat rearrangements, supporting the idea that RecA-independent recombination occurs during the process of chromosome replication [14]. A replication slipped misalignment model [14] proposed that a pause in DNA synthesis and dissociation of the polymerase from its template allows the nascent strand to translocate to a new pairing position. Slipped misalignment is thought to occur on single-stranded DNA and thus more frequently during lagging-strand synthesis [14]. The availability of single-stranded DNA on the lagging-strand template, and thus, the length of the Okazaki fragments, might constitute parameters that govern the efficiency of the deletion events and might explain the strong dependence of the deletion frequencies on the proximity of the repeated sequences [14]. Our findings that the distance between the repeats in the 1–11 kb range has no influence on the deletion frequency raise the question as to whether these events were generated through a slipped misalignment mechanism, thus implying the presence of very large single stranded DNA regions on the lagging strand template in *D*. *radiodurans*. A response to this question awaits better knowledge of the replication machinery in *D*. *radiodurans* and a determination of the average size of Okazaki fragments in this bacterium.

### Involvement of single strand annealing in RecA-independent recombination between chromosomal direct repeats

We found that the frequencies of the deletion events in Δ*recA* Δ*ddrB* (or Δ*recO* Δ*ddrB*) bacteria were reduced 5-fold and 18- to 20-fold as compared with those measured in the single Δ*recA* (or Δ*recO*) mutant counterparts (Figs 1B and 5) in strains containing repeats separated by 1479 bp and 3,500 to 10,500 bp, respectively. These results strongly suggest that the DdrB protein strongly stimulates RecA-independent recombination in *D*. *radiodurans*. The DdrB protein was shown to bind single stranded DNA [5] and to mediate *in vitro* fast annealing of complementary oligomers [6]. *In vivo*, the single strand annealing activity of DdrB is supported by its involvement in plasmid establishment during natural transformation [4]. Thus, we propose that RecA-independent recombination between direct repeats occurs mainly through a DdrB-dependent single strand annealing (SSA) pathway. SSA was first proposed to explain circularization of linear duplex phage DNA containing terminal repetitions by annealing complementary terminal single overhangs [31]. The SSA model was postulated later to take place in eukaryotic cells [32,33] where it is facilitated by RPA and RAD52 in a RAD51-independent manner [34,35]. SSA involves an initial DNA double strand break in the sequence between the duplications followed by the action of a 5’ to 3’ exonuclease to expose single stranded regions in both repeats that are subsequently aligned and annealed by the RAD52-RPA-ssDNA ternary complex. Annealed intermediates are then processed by digestion of the displaced single stranded DNA, polymerase filling-in and ligation to generate the final recombination product (For review, see [36]).

Although DdrB does not share sequence similarity with the eukaryotic RAD52 protein, it might act as its functional equivalent [37,38]. The activity of the RAD52 protein is strongly stimulated by the presence of RPA [39,40]. In contrast, the single strand annealing activity of DdrB is not stimulated but rather inhibited by inclusion of the SSB protein in the *in vitro* annealing reaction [4,6]. The deinococcal SSB protein is an essential protein and the DdrB protein is unable, even when overexpressed, to replace SSB for cell viability [41]. SSB is crucial for all aspects of DNA metabolism [42] while DdrB seems to have a more specialized role in DNA repair and plasmid transformation by stimulating the SSA pathway. Both DdrB and SSB bind to 3’ single stranded tails of resecting ends [5]. The ends can be engaged in two alternative pathways: annealing to complementary ssDNA in the SSA pathway, or, depending on the formation of a RecA nucleofilament, invasion of homologous dsDNA to promote strand exchange in homologous recombination or to prime DNA synthesis in ESDSA. Polymerization of RecA on ssDNA requires the displacement of SSB or DdrB from ssDNA. The SSB protein can be efficiently displaced through the action of RecO and RecR proteins [43,44]. DdrB protein binds more tightly than SSB to ssDNA [5] and might be displaced with more difficulty from ssDNA. We propose that, in *D*. *radiodurans*, homologous recombination and SSA might also compete for substrate in making deletions between direct repeats. Reams *et al*. [45] proposed that, in *Salmonella enterica*, a single-strand annealing pathway might also be activated to generate duplication between tandem copies of the ribosomal RNA genes (*rrn)* when two single-stranded DNA ends are provided and neither strand is coated with inhibitory RecA protein. Under these conditions, the activation of single-strand annealing might compensate the loss of homologous recombination [45].

### A single strand annealing mechanism is involved in the rescue of stalled replication fork in Δ*recA* bacteria

We found that Δ*recA* (or Δ*recO*) bacteria had a 15 fold decreased plating efficiency as compared with the wild type during exponential growth phase (Fig 4). This important lethal sectoring suggests that problems resulting in the arrest of replication fork (see for review [46]) occur at high frequency in *D*. *radiodurans*, and that RecA-mediated recombination plays a key role in the recovery of stalled replication forks in this bacterium. A further (2-fold) decreased plating efficiency was observed when a DdrB deficiency was combined with a RecA (or RecO) deficiency (Fig 4). These results are consistent with a single strand annealing model but also with any model that envisions annealing of complementary DNA strands, for example misannealing of the direct repeats during recovery from replication fork collapse in cells devoid of the RecA protein. *D*. *radiodurans* also seems to be very sensitive to prolonged stationary phase, with a rapid loss of cell viability when proteins involved in homologous recombination were absent, suggesting that DNA double strand breaks were generated in “old” cells and could not be repaired in the absence of RecA or RecO proteins.

### Absence of UvrD increases the frequency of recombination between the repeats

Another important feature was the increased recombination frequency between repeated sequences measured in a *D*. *radiodurans* mutant devoid of the UvrD protein at a level almost equivalent to those measured after irradiation of wild type bacteria (Fig 6). It was previously shown that *uvrD* mutations enhance tandem repeat deletion in the *E*. *coli* chromosome [23,47] and stimulate RecA-dependent recombination [21,48,49]. In *E*. *coli*, mutations in *uvrD* induce the SOS response, a common phenotype in cells with replication defects [50]. The obstacles possibly encountered by replication forks during their progression are multiple, such as tightly bound proteins, nicks or DNA lesions. The Rep helicase acts by dislodging proteins in front of replication forks [51–53] and its absence results in a marked slowing down of replication progression [54], suggesting increased fork arrest. Simultaneous inactivation of Rep and UvrD helicases is lethal in *E*. *coli* [23] suggesting that UvrD might partially substitute for the Rep protein in ensuring replication progression [55]. In favour of this hypothesis, it was recently shown that UvrD displaces the obstacles downstream of the replisome *in vitro* [52] and plays a major role to displace transcription complexes [56]. Moreover, it was proposed that UvrD acts at blocked replication forks by clearing RecA, facilitating replication fork reversal [57, 58], a hypothesis supported by the ability of UvrD to directly remove RecA nucleoprotein filaments *in vitro* [25].

In *D*. *radiodurans*, we previously showed that inactivation of *uvrD* results in a marked slowing down of replication progression in un-irradiated cells [3]. During post-irradiation recovery through ESDSA, the absence of UvrD results in a delayed kinetics of DNA double strand break repair that coincided with delayed and less extensive DNA synthesis than that observed in the wild type cells [3]. *D*. *radiodurans* bacteria are naturally devoid of the RecB and RecC proteins, and it was suggested that UvrD, in association with the RecJ exonuclease, might play an important role in the processing of DNA double strand ends required for priming of DNA synthesis, but also may act in the DNA synthesis elongation step of ESDSA and more generally may play an important role for the progression of replication forks [3]. It is important to notice that we were unable to obtain mutants devoid of the RecJ protein [3], and *recJ* mutants constructed by Hua and his collaborators were shown to grow very slowly and to be thermosensitive [59].

In *D*. *radiodurans*, we were unable to delete the *recA* gene when bacteria were devoid of the UvrD protein, suggesting colethality of *uvrD* and *recA* deficiencies. These results are reminiscent of phenotypes observed in particular *rad3* mutants of *Saccharomyces cerevisiae*. The RAD3 gene, a homolog of the human gene XPD, encodes a helicase which is a component of the NER apparatus as part of the transcription factor TFIIH. Interestingly *rad3-101* and *rad3-102* mutants accumulate DNA double strand breaks and are lethal when in combination with mutations in recombinational repair genes, strongly suggesting that Rad3 protein influences either the generation of DNA double strand breaks or their processing by homologous recombination [60].

We propose that the absence of UvrD in *D*. *radiodurans* may disturb the progression of the replication fork, and thus might, as RAD3 in *S*. *cerevisiae*, influence the generation of DNA double strand break, favouring recombination and also single strand annealing between DNA repeats.

### Induction of recombination between direct repeats by ionizing radiation

We used our assay to analyze the impact of the presence of repeated sequence on the stability of the genome in γ-irradiated cells. We found that exposure to a dose of 5 kGy γ-irradiation increased the recombination level about 10-fold in the wild type but to a lesser extent in cells devoid of the DdrB protein, suggesting that SSA might play an important role in recombination between the duplicated sequences during the process of genome reconstitution.

In *D*. *radiodurans*, interplasmidic recombination between homologous regions was previously shown to be induced by exposure to γ-radiation [61]. Moreover, when two Tet^S^ alleles were inserted on the same chromosome into two randomly distant sites, 2% of Tet^R^ bacteria were found among the surviving cells exposed to 17.5 kGy, whereas Tet^R^ isolates were only very rarely found without irradiation [62]. Interestingly, when two slightly different *E*. *coli* plasmids were inserted in the *D*.*radiodurans* genome generating adjacent duplication insertions, circular derivatives of the tandemly integrated plasmids were formed in the first 1.5 h postirradiation before the onset of *recA*-dependent repair in cells exposed to 17.5 kGy γ-irradiation. These circular derivatives had structures consistent with the hypothesis that DNA repair occurred immediately postirradiation by a *recA*-independent single strand annealing process [63]. These authors proposed that SSA may be a preparatory step for further DNA repair in wild-type *D*. *radiodurans*, a hypothesis in accordance with our recent results, suggesting that DdrB-dependent single-strand annealing might facilitate the assembly of the myriad of small fragments generated by extreme radiation exposure to generate suitable substrates for subsequent ESDSA-promoted genome reconstitution [4]. Genome reassembly in irradiated *D*. *radiodurans* cells was considered for a long time as an error-free process since no genome rearrangements were detected after post-irradiation DNA repair. Gross chromosomal rearrangements were detected for the first time in *recA* ^+^ *D*. *radiodurans* cells exposed to extremely high γ-doses (25 kGy) and in *recA* mutant cells that survived 5 kGy γ-radiation [64]. The *recA* mutants were also shown to be prone to spontaneous DNA rearrangements during normal exponential growth [64]. These authors presumed that SSA, by pairing ectopic repetitive sequences, may be the main source of these chromosomal rearrangements, a hypothesis reinforced by our results suggesting an important role of SSA in recombination between repeated sequences (this work), in DNA double strand break repair in cells devoid of the RecA protein [4,6], and in early reassembly of small DNA fragments when cells were exposed to high γ-doses [4].

### Conclusion

Altogether, these results suggest that SSA plays a major role in RecA-independent recombination between repeated sequences in the radioresistant *D*. *radiodurans* bacterium. In un-irradiated wild type bacteria, the deletion events might result, as proposed by Susan Lovett in *E*. *coli*, from RecA-dependent intermolecular unequal crossing over or intramolecular recombination between the overlapping 5’ and 3’ regions of the *tetA* gene, and from RecA-independent processes such as replication slippage or template switching [10] or single strand annealing [10,14]. Difficulties in replication can lead to breakage of the fork when replication forks are halted by obstacles or DNA damage in virtually every cell and every cell generation [65,66]. If this occurs in the context of repeated DNA sequences, single-stranded DNA substrates might be generated by resection of the DNA ends, and genetic rearrangements can result through strand-invasion of the broken chromosome with its sister or through SSA at the repeats. Replication of damaged DNA templates can further elevate the probability of fork breakage [67,68]. Moreover, when *D*. *radiodurans* cells were exposed to a dose of 5 kGy γ-irradiation, generating hundreds DNA double strand breaks, DdrB-dependent SSA and RecA-dependent ESDSA processes involved in DNA double strand break repair increased the opportunities to generate deletion events when DNA repeats are present in the DNA fragments.

## Materials and Methods

### Culture, media

*D*. *radiodurans* strains were grown at 30°C, 150 rpm in TGY2X (1% tryptone, 0.2% dextrose, 0.6% yeast extract) or plated on TGY1X containing 1.5% agar. *E*. *coli* strains were grown at 37°C, 150 rpm in Lysogeny Broth (LB). When necessary, media were supplemented with the appropriate antibiotics used at the following final concentrations: kanamycin, 6 μg/mL; chloramphenicol, 3.5 μg/mL; hygromycin, 50 μg/mL; spectinomycin, 75 μg/mL; tetracycline 2.5 μg/mL for *D*. *radiodurans* and kanamycin, 25 μg/mL or spectinomycin 40 μg/mL for *E*. *coli*.

### Bacterial strains and plasmids

The bacterial strains and plasmids used in this study are listed in Table 1. The *E*. *coli* strains used were DH5α as the general cloning host, and SCS110, a *dam dcm* mutant strain, to propagate plasmids prior introduction into *D*. *radiodurans* via transformation [69 *]*. Transformation of *D*. *radiodurans* with genomic DNA, PCR products, or plasmid DNA was performed as described [70]. All *D*. *radiodurans* strains were derivatives of strain R1 ATCC 13939. The genetic structure and the purity of the mutant strains were checked by PCR. Oligonucleotides used for strain constructions and diagnostic PCR will be provided on request.

**Table 1:** Bacterial strains and plasmids.

Bacterial strains	Description	Source or reference
***E*. *coli***		
DH5α	*supE44 ΔlacU169* (φ80*lacZΔ*M15) *hsdR17 recA1endA1 gyrA96 thi-1 relA1*	Laboratory stock
SCS110	*endA dam dcm supE44*Δ(*lac-proAB*) (*F’- traD36 proABlacI* ^*q*^ *ZΔM15*)	Laboratory stock
***D*. *radiodurans***		
GY9613	R1 ATCC 13939	Laboratory stock
GY12958	as R1 but Δ*radA*Ω*cat*	This work
GY12965	as R1 but Δ*recF*Ω*cat*	[3]
GY12966	as R1 but Δ*recO*Ω*hph*	[3]
GY12967	as R1 but Δ*recR*Ω*cat*	[3]
GY12968	as R1 but Δ*recA*Ω*kan*	[3]
GY12977	as R1 but Δ*mutS1*Ω*cat*	This work
GY13915	as R1 but Δ*ddrB*Ωcat	This work
GY15123	as R1 but Δ*uvrD*Ω*kan*	This work
GY15125	as R1 but Δ*recO*Ω*kan*	This work
GY15180	as R1 but Δ*recA*Ω*cat*	This work
GY16004	as R1 but Δ*uvrD*Ωcat	This work
GY16262	as R1 but Δ*recO*03A9*cat*	This work
GY16626	as GY13915 but Δ*recA*Ω*kan*	This work
GY16636	as GY13915 but Δ*recO*Ω*kan*	This work
GY12947	*amyE’*::P_*groE*_::*tetA*::*’amyE*	This work
GY12949^(a)^	*amyE*’:: (P_groE_:: 5’*tetA*::Pout:: spc_1,479_::3’*tetA*)::’*amyE*	This work
GY15184	as GY12949Δ*recA*Ω*cat*	This work
GY12956	as GY12949 but Δ*radA*Ω*cat*	This work
GY12971	as GY12956 but Δ*recA*Ω*kan*	This work
GY12978	as GY12949 but Δ*mutS1*Ω*cat*	This work
GY16620	as GY12978 but Δ*recA*Ω*kan*	This work
GY12950^(b)^	as GY12949 but 1 mutation C420T in 5’ *tetA’*	This work
GY12980	as GY12950 but Δ*mutS1*Ω*cat*	This work
GY16624	as GY12950 but Δ*recA*Ω*kan*	This work
GY16618	as GY12980 but Δ*recA*Ω*kan*	This work
GY12948^(b)^	as GY12949 but 3 mutations G410A, A620G, delA767 in *tetA’*	This work
GY12979	as GY12948 but Δ*mutS1*Ω*cat*	This work
GY16622	as GY12948 but Δ*recA*Ω*kan*	This work
GY16616	as GY12979 but Δ*recA*Ω*kan*	This work
GY12963	as GY12949 but Δ*recO*Ω*hph*	This work
GY12955	as GY12949 but Δ*recF*Ω*cat*	This work
GY12953	as GY12949 but Δ*uvrD*Ωcat	This work
GY16016	as GY12949 but Δ*ddrB*Ωcat	This work
GY16628	as GY16016 but Δ*recA*Ω*kan*	This work
GY16638	as GY16016 but Δ*recO*Ω*kan*	This work
GY16209^(a)^	*amyE*’:: (PgroE:: 5’*tetA*::P_out_:: spc_3,500_::3’*tetA*)::’*amyE*	This work
GY16238	as GY16209 but Δ*recA*Ω*cat*	This work
GY16264	as GY16209 but Δ*recF*Ω*cat*	This work
GY16262	as GY16209 but Δ*recO*Ω*cat*	This work
GY16608	as GY16209 but Δ*uvrD*Ω*cat*	This work
GY16268	as GY16209 but Δ*ddrB*Ω*cat*	This work
GY16630	as GY16268 but Δ*recA*Ω*kan*	This work
GY16640	as GY16628 but Δ*recO*Ω*kan*	This work
GY16227^(a)^	*amyE’*:: (P_*groE*_:: 5’ *tetA*::P_out_:: *spc*_3,500_:: *hph*_3000_:: 3’tetA)::’*amyE*	This work
GY16244	as GY16227but Δ*recA*Ω*cat*	This work
GY16276	as GY16227 but Δ*recF*Ω*cat*	This work
GY16278	as GY16227 but Δ*recO*Ω*cat*	This work
GY16612	as GY16227 but Δ*uvrD*Ω*cat*	This work
GY16282	as GY16227 but Δ*ddrB*Ω*cat*	This work
GY16632	as GY16282 but Δ*recA*Ω*kan*	This work
GY16642	as GY16282 but Δ*recO*Ω*kan*	This work
GY16211^(a)^	*amyE*’:: (PgroE:: 5’*tetA*::P_out_:: *spc*_4,500_::3’*tetA*)::’*amyE*	This work
GY16235^(a)^	*amyE*’:: (PgroE::5’tetA::P_out_::*spc*_4,500_::*hph*_6000_::3’*tetA*)::’*amyE*)	This work
GY16252	as GY16235 but Δ*recA*Ω*cat*	This work
GY16292	as GY16235 but Δ*recF*Ω*cat*	This work
GY16290	as GY16235 but Δ*recO*Ω*cat*	This work
GY16614	as GY16235 but Δ*uvrD*Ω*cat*	This work
GY16296	as GY16235 but *ddrB*Ω*cat*	This work
GY16634	as GY16296 but Δ*recA*Ω*kan*	This work
GY16644	as GY16296 but Δ*recO*Ω*kan*	This work
GY15102^(a)^	*amyE’*::(*P*_*groE*_::*5’tetA’*::*P*_*kat*_::*hph)*::*’amyE*	This work
GY15147	GY15102 (p15002)	This work
GY15149	as GY15147 but Δ*radA*Ω*cat*	This work
GY15156	as GY15147 but Δ*uvrD*Ω*kan*	This work
GY15158	as GY15147 but Δ*recA*Ω*cat*	This work
GY15160	as GY15147 but Δ*recF*Ω*cat*	This work
**Plasmids**		
p11615	Source of *tetA* cassette in *D*. *radiodurans*	[71]
p11086	Source of *kan* cassette in *D*. *radiodurans*	Laboratory stock
p11520	Shuttle vector *E*. *coli* / *D*. *radiodurans*, *spec*	[72]
p11554	Deletion of the *cat* gene in p11520, shuttle vector *E*. *coli* / *D*. *radiodurans*, *spec*	Laboratory stock
p13025	pUC18 derivative encoding *lacZ*	[73]
p12625	pUC19 containing *hph* and *D*. *radiodurans katA* promoter (alias pKatHPH4)	[74]
p15002^(a)^	3*’tetA* inserted in p11554 between StuI and BamHI restriction sites	This work
P15070	Insertion between HindIII and XbaI sites of p13025 of a PCR fragment containing the *P*_*kat*_::*hph* gene amplified from p12625	This work

^(a)^ 5’*tetA* and the 3’*tetA* contain the 1–779 and the 342–1190 part of the *tetA* coding region, respectively. They contain a 342–779 overlapping region. The positions are numbered relatively to the first base of the initiation codon of *tetA*.

^(b)^ The positions of the mutations in the 5’*tetA* fragment are numbered relatively to the first base of the initiation codon of *tetA*.

#### Construction of tester strains for recombination assays

The strain GY12947 was constructed by the integration of the tetA gene into the dispensable amyE (DR_1472) gene by transformation of GY9613 (ATCC 13939 R1 strain) by the tripartite ligation product containing the 5’ and 3’parts of amyE and the tetA gene amplified by PCR using GY9613 genomic DNA and plasmid p11615 as template, respectively.

GY12947 was then used to amplify the 778 bp of the 5’ part (called 5’*tetA*) and the 850 bp of the 3’ part (called 3’*tetA*) of the coding region of the 1,190 bp *tetA* gene. The 5’*tetA* and 3’*tetA* fragments contain 438 bp overlapping sequences. Tripartite ligation products containing the 5’*tetA* and 3’*tetA* fragments separated by spacers of 1,479, 3,500 or 4,500 bp containing a *spec* gene amplified by PCR from plasmid p11554 were used to transform GY12947 giving rise to GY12949, GY16209, GY16211, respectively. Independent clones were saved for each strain and constructions were verified by DNA sequencing. Mutations generated during PCR amplification of 5‘*tetA* using GY12947 as template were detected in the 438 bp overlapping sequence of the 5‘*tetA* in two clones of GY12949 and the strains containing mutations in the *5’tetA* overlapping region were renamed GY12948 (3 mutations) and GY12950 (1 mutation).

Strains with larger spacers between the *tetA* overlapping regions were obtained by transformation of GY16209 and GY16211 with a tripartite ligation product of a 3,000 or 6,000 bp PCR fragment containing the *hph* gene amplified from plasmid p15070, and 5’ and 3’ regions of the *spec* gene amplified by PCR from GY12949 yielding GY16227 containing a 6,500 bp spacer and GY16235 containing a 10,500 bp spacer, respectively. The right integration of spacers, and the purity of the strains were checked by PCR. The *5’tetA*, the *3’tetA* and the intervening sequences were checked by DNA sequencing.

For the recombination assays between chromosomal and plasmid DNA, strain GY15102, in which the 5’ region of the *tetA* gene (5’*tetA*) and an *hph* cassette were inserted in the *amyE* gene, was transformed by the plasmid p15002 obtained by cloning in plasmid p11554 of the 3’*tetA* region to give rise to strain GY15147. Plasmid and chromosomal DNA contained the same 438 bp homologous sequences as those present in the genomic DNA of strains used for measurement of inter- or intra-chromosomal recombination.

Deletion mutant derivatives of all the recombination tester strains were obtained by transformation of the tester strains with genomic DNA of Δ*recA*, Δ*mutS*, Δ*uvrD*, Δ*recF*, Δ*recO*, Δ*ddrB*, Δ*radA* derivatives of R1 strain, available in the laboratory or constructed by the tripartite ligation method [71]. The Δ*recA*Δ*radA*, Δ*recA*Δ*mutS*, Δ*recA*Δ*ddrB*, Δ*recO*Δ*ddrB* double mutants were obtained by transformation of the single Δ*radA*, Δ*mutS* or Δ*ddrB* mutants by genomic DNA of Δ*recA or* Δ*recO* bacteria. Δ*uvrD* bacteria were also transformed by genomic DNA of Δ*recA*, Δ*recF and* Δ*recR* bacteria. All strains were extensively purified on selective medium to obtain homogenotization of the deletions before to be checked by PCR for their purity. Attempts to obtain homogenotes containing the *ΔuvrD*Δ*recA* allele, the *ΔuvrD*Δ*recF* allele and the *ΔuvrD*Δ*recR* allele on each of the multiple genomic copies present in *D*. *radiodurans* were unsuccesful, even after extensive purification on selective plates (see Results section).

### Inter- or intra-chromosomal recombination

Cells were plated on TGY agar and incubated at 30°C for 3 days, or 5 days for Δ*recA*, Δ*recO* and Δ*recF* mutant bacteria and 7 days for double mutant Δ*ddrB* Δ*recA* and Δ*ddrB* Δ*recO* bacteria. Three to six colonies per strain were inoculated in 3 mL of TGY2X and incubated at 30°C, 150 rpm. Appropriate dilutions of the bacterial cultures, grown to OD_650nm_ = 1.5 were plated on TGY and TGY + tetracycline 2.5 μg/mL. Colonies were counted after 4 to 7 days of incubation at 30°C. The experiments were repeated at least three times, using, when possible, strain isolates obtained independently during the strain constructions.

To measure recombination between repeated sequences after γ-irradiation, bacterial strains were treated as previously described upstream, except that bacterial cultures were grown to an OD_650nm_ = 0.5 before being concentrated by centrifugation in TGY2X to an OD_650nm_ = 10 and irradiated on ice at a dose of 5 kGy with a ^60^Co irradiation system (LABRA, CEA, Saclay) at a dose rate of 100 Gy/min. Following irradiation, samples of 100 μL were inoculated in 4.9 mL of TGY2X and incubated at 30°C, 150 rpm. After 20 hours of post-irradiation incubation, appropriate dilutions of bacterial culture were plated on TGY and TGY + tetracycline 2.5 μg/mL. Colonies were counted after 4-7days of incubation at 30°C. Unirradiated controls were treated as irradiated cells, except that they were maintained on ice without irradiation during the period when the irradiated cells were exposed to γ-rays.

### Recombination between chromosomal and plasmid DNA

Cells containing plasmid p15002 were plated on TGY agar + spectinomycin (75 μg/mL) and incubated at 30°C during 3 days (or 5 days for Δ*recA* and Δ*recF* bacteria). Three colonies per strain were inoculated in 3 mL of TGY2X + spectinomycin (75 μg/mL) and incubated at 30°C, 150 rpm. Appropriate dilutions of the bacterial cultures grown to OD_650nm_ = 1.5 were plated on TGY and TGY+ tetracycline 2.5 μg/mL. Colonies were counted after 4 to 7 days of incubation at 30°C.

### Growth rate and lethal sectoring of Δ*ddrB* Δ*recA and* Δ*ddrB* Δ*recO* double mutants

Strains were streaked on TGY plates supplemented with the appropriate antibiotics. Independent colonies were inoculated in 3 mL TGY2X supplemented with the appropriate antibiotics (only kanamycin for double mutants) and grown at 30°C to an OD_650nm_ = 1.5. Cultures were then diluted 200 to 5,000 fold and grown overnight at 30°C to an OD_650nm_ = 0.1 (time 0 of the growth curves). Then, the OD_650nm_ was measured and appropriate dilutions were plated on TGY plates at different times during 80 h of incubation at 30°C with agitation (150 rpm). Colonies were counted after 3 (WT or Δ*ddrB* bacteria), 5 (Δ*recA* or Δ*recO* bacteria) or 7 days (Δ*ddrB* Δ*recA* or Δ*ddrB* Δ*recO* bacteria) of incubation at 30°C.

### Statistical analysis

In Figs 1 and 3 and 5, in order to establish the statistical differences between the [Tet^R^] frequencies measured in mutant and WT strains, non parametric Dunn’s multiple comparison test [75] were used, taking into account the p-value correction and performed with the GraphPad Prism6 software. All the comparisons were bi-sided.

Linear regressions and the slope significances observed in S2 Fig were estimated using the GraphPad Prism6 software.

In Fig 6, statistically significant differences between the irradiated and the non-irradiated conditions were calculated by non-parametric Mann-Withney tests performed in GraphPad Prism 6 software.

## Supporting Information

S1 Fig Integration of plasmid DNA in chromosomal DNA verified by PCR in [Tet^R^] bacteria. **A.** Schematic representation of the position of the primer pairs a and b, a and d, c and d, and e and f, used to test the integration of the plasmid in chromosomal DNA **B.** GY15147 bacteria containing a 5’*tetA* region inserted in the *amyE* gene and a 3’*tetA* region carried by plasmid p15002 were plated on TGY-Agar plates with or without tetracycline. The genomic DNA of 3 independent [Tet^R^] colonies picked and purified on TGY-Agar plates containing 2.5 μg / mL tetracycline and 1 [Tet^S^] colony picked on TGY-Agar plates was purified and used as templates to verify by PCR the integration of the plasmid using the primers pairs described in S1A. Purified p15002 plasmid DNA and genomic DNA of strain GY15102 that does not contain plasmid p15002 were used as controls. PCR fragment sizes are presented for each DNA template. The fragment of 9812 bp was not amplified under the conditions we used for PCR amplification.
(PDF)

S2 Fig Linear regression models of the evolution of the [Tet^R^] bacteria frequencies as a function of the number of base pair changes in one of the repeat in WT, Δ*recA*, Δ*mutS* and Δ*mut* Δ*recA* strains. (PDF)

S3 Fig Non-homogenotization of Δ*recA*, Δ*recF* and Δ*recR* deletions in Δ*uvrD* bacteria. **A.** Diagnostic PCRs for the deletion of *recA* and *uvrD* genes. **B.** Diagnostic PCRs for the deletion of *recF* and *uvrD* genes. **C.** Diagnostic PCRs for the deletion of *recR* and *uvrD* genes. Schematic allelic replacement, primers and PCR fragment sizes are represented for each gene.
(PDF)

S4 Fig Homogenotization of Δ*recA* and Δ*recO* deletions in Δ*ddrB* bacteria. **A.** Diagnostic PCRs for the deletion of *recA* and *ddrB* genes. **B.** Diagnostic PCRs for the deletion of *recO* and *ddrB* genes. Schematic allelic replacement, primers and PCR fragment sizes are represented for each gene.
(PDF)

## References

[1] Zahradka K, Slade D, Bailone A, Sommer S, Averbeck D, et al (2006) Reassembly of shattered chromosomes in *Deinococcus radiodurans* . Nature 443: 569–573. 17006450 10.1038/nature05160

[2] Slade D, Lindner AB, Paul G, Radman M (2009) Recombination and replication in DNA repair of heavily irradiated *Deinococcus radiodurans* . Cell 136: 1044–1055. 10.1016/j.cell.2009.01.018 19303848

[3] Bentchikou E, Servant P, Coste G, Sommer S (2010) A major role of the RecFOR pathway in DNA double-strand-break repair through ESDSA in *Deinococcus radiodurans* . PLoS Genet 6: e1000774 10.1371/journal.pgen.1000774 20090937 PMC2806897

[4] Bouthier de la Tour C, Boisnard S, Norais C, Toueille M, Bentchikou E, et al (2011) The deinococcal DdrB protein is involved in an early step of DNA double strand break repair and in plasmid transformation through its single-strand annealing activity. DNA Repair (Amst) 10: 1223–1231. 21968057 10.1016/j.dnarep.2011.09.010 PMC3268515

[5] Norais CA, Chitteni-Pattu S, Wood EA, Inman RB, Cox MM (2009) An alternative *Deinococcus radiodurans* SSB induced by ionizing radiation: The DdrB protein. J Biol Chem. 10.1074/jbc.M109.010454 PMC2755865 19515845

[6] Xu G, Lu H, Wang L, Chen H, Xu Z, et al (2010) DdrB stimulates single-stranded DNA annealing and facilitates RecA-independent DNA repair in *Deinococcus radiodurans* . DNA Repair (Amst) 9: 805–812. 20451472 10.1016/j.dnarep.2010.04.006

[7] Mazin AV, Kuzminov AV, Dianov GL, Salganik RI (1991) Mechanisms of deletion formation in *Escherichia coli* plasmids. II. Deletions mediated by short direct repeats. Mol Gen Genet 228: 209–214. 1679526 10.1007/BF00282467

[8] Dianov GL, Kuzminov AV, Mazin AV, Salganik RI (1991) Molecular mechanisms of deletion formation in *Escherichia coli* plasmids. I. Deletion formation mediated by long direct repeats. Mol Gen Genet 228: 153–159. 1679524 10.1007/BF00282460

[9] Bi X, Liu LF (1994) *recA*-independent and *recA*-dependent intramolecular plasmid recombination. Differential homology requirement and distance effect. J Mol Biol 235: 414–423. 8289271 10.1006/jmbi.1994.1002

[10] Lovett ST, Gluckman TJ, Simon PJ, Sutera VA Jr., Drapkin PT (1994) Recombination between repeats in *Escherichia coli* by a *recA*-independent, proximity-sensitive mechanism. Mol Gen Genet 245: 294–300. 7816039 10.1007/BF00290109

[11] Bierne H, Vilette D, Ehrlich SD, Michel B (1997) Isolation of a *dnaE* mutation which enhances RecA-independent homologous recombination in the *Escherichia coli* chromosome. Mol Microbiol 24: 1225–1234. 9218771 10.1046/j.1365-2958.1997.4381795.x

[12] Saveson CJ, Lovett ST (1997) Enhanced deletion formation by aberrant DNA replication in *Escherichia coli* . Genetics 146: 457–470. 9177997 10.1093/genetics/146.2.457 PMC1207988

[13] Saveson CJ, Lovett ST (1999) Tandem repeat recombination induced by replication fork defects in *Escherichia coli* requires a novel factor, RadC. Genetics 152: 5–13. 10224240 10.1093/genetics/152.1.5 PMC1460591

[14] Michel B (2000) Replication fork arrest and DNA recombination. Trends Biochem Sci 25: 173–178. 10754549 10.1016/s0968-0004(00)01560-7

[15] Lovett ST (2004) Encoded errors: mutations and rearrangements mediated by misalignment at repetitive DNA sequences. Mol Microbiol 52: 1243–1253. 15165229 10.1111/j.1365-2958.2004.04076.x

[16] Bzymek M, Lovett ST (2001) Evidence for two mechanisms of palindrome-stimulated deletion in *Escherichia coli*: single-strand annealing and replication slipped mispairing. Genetics 158: 527–540. 11404319 10.1093/genetics/158.2.527 PMC1461685

[17] Nguyen HH, de la Tour CB, Toueille M, Vannier F, Sommer S, et al (2009) The essential histone-like protein HU plays a major role in *Deinococcus radiodurans* nucleoid compaction. Mol Microbiol 73: 240–252. 10.1111/j.1365-2958.2009.06766.x 19570109

[18] Meima R, Lidstrom ME (2000) Characterization of the minimal replicon of a cryptic *Deinococcus radiodurans* SARK plasmid and development of versatile *Escherichia coli-D*. *radiodurans* shuttle vectors. Appl Environ Microbiol 66: 3856–3867. 10966401 10.1128/aem.66.9.3856-3867.2000 PMC92231

[19] Beam CE, Saveson CJ, Lovett ST (2002) Role for *radA*/*sms* in recombination intermediate processing in *Escherichia coli* . J Bacteriol 184: 6836–6844. 12446634 10.1128/JB.184.24.6836-6844.2002 PMC135464

[20] Cooper DL, Boyle DC, Lovett ST (2015) Genetic analysis of *Escherichia coli* RadA: functional motifs and genetic interactions. Mol Microbiol 95: 769–779. 10.1111/mmi.12899 25484163 PMC4357459

[21] Konrad EB (1977) Method for the isolation of *Escherichia coli* mutants with enhanced recombination between chromosomal duplications. J Bacteriol 130: 167–172. 323226 10.1128/jb.130.1.167-172.1977 PMC235189

[22] Feinstein SI, Low KB (1986) Hyper-recombining recipient strains in bacterial conjugation. Genetics 113: 13–33. 3519362 10.1093/genetics/113.1.13 PMC1202792

[23] Washburn BK, Kushner SR (1991) Construction and analysis of deletions in the structural gene (*uvrD*) for DNA helicase II of *Escherichia coli* . J Bacteriol 173: 2569–2575. 1849510 10.1128/jb.173.8.2569-2575.1991 PMC207822

[24] Morel P, Hejna JA, Ehrlich SD, Cassuto E (1993) Antipairing and strand transferase activities of *E*. *coli* helicase II (UvrD). Nucleic Acids Res 21: 3205–3209. 8341594 10.1093/nar/21.14.3205 PMC309756

[25] Veaute X, Delmas S, Selva M, Jeusset J, Le Cam E, et al (2005) UvrD helicase, unlike Rep helicase, dismantles RecA nucleoprotein filaments in *Escherichia coli* . EMBO J 24: 180–189. 15565170 10.1038/sj.emboj.7600485 PMC544901

[26] Cox MM, Battista JR (2005) *Deinococcus radiodurans*—the consummate survivor. Nat Rev Microbiol 3: 882–892. 16261171 10.1038/nrmicro1264

[27] Treangen TJ, Abraham AL, Touchon M, Rocha EP (2009) Genesis, effects and fates of repeats in prokaryotic genomes. FEMS Microbiol Rev 33: 539–571. 19396957 10.1111/j.1574-6976.2009.00169.x

[28] Makarova KS, Aravind L, Wolf YI, Tatusov RL, Minton KW, et al (2001) Genome of the extremely radiation-resistant bacterium *Deinococcus radiodurans* viewed from the perspective of comparative genomics. Microbiol Mol Biol Rev 65: 44–79. 11238985 10.1128/MMBR.65.1.44-79.2001 PMC99018

[29] Marsin S, Mathieu A, Kortulewski T, Guerois R, Radicella JP (2008) Unveiling novel RecO distant orthologues involved in homologous recombination. PLoS Genet 4: e1000146 10.1371/journal.pgen.1000146 18670631 PMC2475510

[30] Bruand C, Bidnenko V, Ehrlich SD (2001) Replication mutations differentially enhance RecA-dependent and RecA-independent recombination between tandem repeats in *Bacillus subtilis* . Mol Microbiol 39: 1248–1258. 11251841 10.1111/j.1365-2958.2001.02312.x

[31] Thomas CA (1967) The recombination of DNA molecules In: press TRU, editor. The Neurosciences A study program. New York pp. 162–182.

[32] Lin FL, Sperle K, Sternberg N (1984) Model for homologous recombination during transfer of DNA into mouse L cells: role for DNA ends in the recombination process. Mol Cell Biol 4: 1020–1034. 6330525 10.1128/mcb.4.6.1020-1034.1984 PMC368869

[33] Mezard C, Nicolas A (1994) Homologous, homeologous, and illegitimate repair of double-strand breaks during transformation of a wild-type strain and a rad52 mutant strain of *Saccharomyces cerevisiae* . Mol Cell Biol 14: 1278–1292. 8289807 10.1128/mcb.14.2.1278-1292.1994 PMC358483

[34] Grimme JM, Honda M, Wright R, Okuno Y, Rothenberg E, et al (2010) Human Rad52 binds and wraps single-stranded DNA and mediates annealing via two hRad52-ssDNA complexes. Nucleic Acids Res 38: 2917–2930. 10.1093/nar/gkp1249 20081207 PMC2875008

[35] Rothenberg E, Grimme JM, Spies M, Ha T (2008) Human Rad52-mediated homology search and annealing occurs by continuous interactions between overlapping nucleoprotein complexes. Proc Natl Acad Sci U S A 105: 20274–20279. 10.1073/pnas.0810317106 19074292 PMC2629295

[36] Paques F, Haber JE (1999) Multiple pathways of recombination induced by double-strand breaks in *Saccharomyces cerevisiae* . Microbiol Mol Biol Rev 63: 349–404. 10357855 10.1128/mmbr.63.2.349-404.1999 PMC98970

[37] Sugiman-Marangos S, Junop MS (2010) The structure of DdrB from *Deinococcus*: a new fold for single-stranded DNA binding proteins. Nucleic Acids Res 38: 3432–3440. 10.1093/nar/gkq036 20129942 PMC2879517

[38] Sugiman-Marangos SN, Peel JK, Weiss YM, Ghirlando R, Junop MS (2013) Crystal structure of the DdrB/ssDNA complex from *Deinococcus radiodurans* reveals a DNA binding surface involving higher-order oligomeric states. Nucleic Acids Res 41: 9934–9944. 10.1093/nar/gkt759 23975200 PMC3834827

[39] Shinohara A, Shinohara M, Ohta T, Matsuda S, Ogawa T (1998) Rad52 forms ring structures and co-operates with RPA in single-strand DNA annealing. Genes Cells 3: 145–156. 9619627 10.1046/j.1365-2443.1998.00176.x

[40] Sugiyama T, New JH, Kowalczykowski SC (1998) DNA annealing by RAD52 protein is stimulated by specific interaction with the complex of replication protein A and single-stranded DNA. Proc Natl Acad Sci U S A 95: 6049–6054. 9600915 10.1073/pnas.95.11.6049 PMC27583

[41] Lockhart JS, DeVeaux LC (2013) The essential role of the *Deinococcus radiodurans ssb* gene in cell survival and radiation tolerance. PLoS One 8: e71651 10.1371/journal.pone.0071651 23951213 PMC3739723

[42] Shereda RD, Kozlov AG, Lohman TM, Cox MM, Keck JL (2008) SSB as an organizer/mobilizer of genome maintenance complexes. Crit Rev Biochem Mol Biol 43: 289–318. 10.1080/10409230802341296 18937104 PMC2583361

[43] Inoue J, Honda M, Ikawa S, Shibata T, Mikawa T (2008) The process of displacing the single-stranded DNA-binding protein from single-stranded DNA by RecO and RecR proteins. Nucleic Acids Res 36: 94–109. 18000001 10.1093/nar/gkm1004 PMC2248737

[44] Hobbs MD, Sakai A, Cox MM (2007) SSB protein limits RecOR binding onto single-stranded DNA. J Biol Chem 282: 11058–11067. 17272275 10.1074/jbc.M611007200

[45] Reams AB, Kofoid E, Duleba N, Roth JR (2014) Recombination and annealing pathways compete for substrates in making *rrn* duplications in *Salmonella enterica* . Genetics 196: 119–135. 10.1534/genetics.113.158519 24214339 PMC3872179

[46] Atkinson J, McGlynn P (2009) Replication fork reversal and the maintenance of genome stability. Nucleic Acids Res 37: 3475–3492. 10.1093/nar/gkp244 19406929 PMC2699526

[47] Bierne H, Seigneur M, Ehrlich SD, Michel B (1997) *uvrD* mutations enhance tandem repeat deletion in the *Escherichia coli* chromosome via SOS induction of the RecF recombination pathway. Mol Microbiol 26: 557–567. 9402025 10.1046/j.1365-2958.1997.6011973.x

[48] Zieg J, Maples VF, Kushner SR (1978) Recombinant levels of *Escherichia coli* K-12 mutants deficient in various replication, recombination, or repair genes. J Bacteriol 134: 958–966. 350859 10.1128/jb.134.3.958-966.1978 PMC222344

[49] Arthur HM, Lloyd RG (1980) Hyper-recombination in *uvrD* mutants of *Escherichia coli* K-12. Mol Gen Genet 180: 185–191. 7003307 10.1007/BF00267368

[50] Ossanna N, Mount DW (1989) Mutations in *uvrD* induce the SOS response in *Escherichia coli* . J Bacteriol 171: 303–307. 2536658 10.1128/jb.171.1.303-307.1989 PMC209587

[51] Yancey-Wrona JE, Wood ER, George JW, Smith KR, Matson SW (1992) *Escherichia coli* Rep protein and helicase IV. Distributive single-stranded DNA-dependent ATPases that catalyze a limited unwinding reaction *in vitro* . Eur J Biochem 207: 479–485. 1321715 10.1111/j.1432-1033.1992.tb17074.x

[52] Guy CP, Atkinson J, Gupta MK, Mahdi AA, Gwynn EJ, et al (2009) Rep provides a second motor at the replisome to promote duplication of protein-bound DNA. Mol Cell 36: 654–666. 10.1016/j.molcel.2009.11.009 19941825 PMC2807033

[53] Atkinson J, Gupta MK, McGlynn P (2011) Interaction of Rep and DnaB on DNA. Nucleic Acids Res 39: 1351–1359. 10.1093/nar/gkq975 20959294 PMC3045612

[54] Lane HE, Denhardt DT (1975) The *rep* mutation. IV. Slower movement of replication forks in *Escherichia coli rep* strains. J Mol Biol 97: 99–112. 1100854 10.1016/s0022-2836(75)80025-8

[55] Taucher-Sholz G, Abdel-Monem M, Hoffman-Berling H (1983) Function of DNA helicases in *E*. *coli*. In: Cozarelli NR, editor. Mechanisms of DNA replication and recombination: Alan, R. Liss, Inc., New York pp. 65–76.

[56] Boubakri H, de Septenville AL, Viguera E, Michel B (2010) The helicases DinG, Rep and UvrD cooperate to promote replication across transcription units *in vivo* . EMBO J 29: 145–157. 10.1038/emboj.2009.308 19851282 PMC2770101

[57] Flores MJ, Bidnenko V, Michel B (2004) The DNA repair helicase UvrD is essential for replication fork reversal in replication mutants. EMBO Rep 5: 983–988. 15375374 10.1038/sj.embor.7400262 PMC1299159

[58] Flores MJ, Sanchez N, Michel B (2005) A fork-clearing role for UvrD. Mol Microbiol 57: 1664–1675. 16135232 10.1111/j.1365-2958.2005.04753.x

[59] Jiao J, Wang L, Xia W, Li M, Sun H, et al (2012) Function and biochemical characterization of RecJ in *Deinococcus radiodurans* . DNA Repair (Amst) 11: 349–356. 22301370 10.1016/j.dnarep.2011.11.008

[60] Montelone BA, Malone RE (1994) Analysis of the rad3-101 and rad3-102 mutations of Saccharomyces cerevisiae: implications for structure/function of Rad3 protein. Yeast 10: 13–27. 8203147 10.1002/yea.320100103

[61] Daly MJ, Ling O, Minton KW (1994) Interplasmidic recombination following irradiation of the radioresistant bacterium *Deinococcus radiodurans* . J Bacteriol 176: 7506–7515. 8002574 10.1128/jb.176.24.7506-7515.1994 PMC197207

[62] Daly MJ, Minton KW (1995) Interchromosomal recombination in the extremely radioresistant bacterium *Deinococcus radiodurans* . J Bacteriol 177: 5495–5505. 7559335 10.1128/jb.177.19.5495-5505.1995 PMC177357

[63] Daly MJ, Minton KW (1996) An alternative pathway of recombination of chromosomal fragments precedes *recA*-dependent recombination in the radioresistant bacterium *Deinococcus radiodurans* . J Bacteriol 178: 4461–4471. 8755873 10.1128/jb.178.15.4461-4471.1996 PMC178212

[64] Repar J, Cvjetan S, Slade D, Radman M, Zahradka D, et al (2010) RecA protein assures fidelity of DNA repair and genome stability in *Deinococcus radiodurans* . DNA Repair (Amst) 9: 1151–1161. 20817622 10.1016/j.dnarep.2010.08.003

[65] Cox MM, Goodman MF, Kreuzer KN, Sherratt DJ, Sandler SJ, et al (2000) The importance of repairing stalled replication forks. Nature 404: 37–41. 10716434 10.1038/35003501

[66] Kuzminov A (1999) Recombinational repair of DNA damage in *Escherichia coli* and bacteriophage lambda. Microbiol Mol Biol Rev 63: 751–813, table of contents. 10585965 10.1128/mmbr.63.4.751-813.1999 PMC98976

[67] Kuzminov A (1995) Instability of inhibited replication forks in *E*. *coli* . Bioessays 17: 733–741. 7661854 10.1002/bies.950170810

[68] Michel B, Ehrlich SD, Uzest M (1997) DNA double-strand breaks caused by replication arrest. EMBO J 16: 430–438. 9029161 10.1093/emboj/16.2.430 PMC1169647

[69] Meima R, Rothfuss HM, Gewin L, Lidstrom ME (2001) Promoter cloning in the radioresistant bacterium *Deinococcus radiodurans* . J Bacteriol 183: 3169–3175. 11325946 10.1128/JB.183.10.3169-3175.2001 PMC95218

[70] Bonacossa de Almeida C, Coste G, Sommer S, Bailone A (2002) Quantification of RecA protein in *Deinococcus radiodurans* reveals involvement of RecA, but not LexA, in its regulation. Mol Genet Genomics 268: 28–41. 12242496 10.1007/s00438-002-0718-x

[71] Mennecier S, Coste G, Servant P, Bailone A, Sommer S (2004) Mismatch repair ensures fidelity of replication and recombination in the radioresistant organism *Deinococcus radiodurans* . Mol Genet Genomics 272: 460–469. 15503140 10.1007/s00438-004-1077-6

[72] Bentchikou E, Servant P, Coste G, Sommer S (2007) Additive effects of SbcCD and PolX deficiencies in the in vivo repair of DNA double strand breaks in *Deinococcus radiodurans* . J Bacteriol. 10.1128/JB.00452-07 PMC1913444 17483232

[73] Devigne A, Ithurbide S, Bouthier de la Tour C, Passot F, Mathieu M, et al (2015) DdrO is an essential protein that regulates the radiation desiccation response and the apoptotic-like cell death in the radioresistant *Deinococcus radiodurans* bacterium. Mol Microbiol. 10.1111/mmi.12991 25754115

[74] Satoh K, Kikuchi M, Ishaque AM, Ohba H, Yamada M, et al (2012) The role of *Deinococcus radiodurans* RecFOR proteins in homologous recombination. DNA Repair (Amst) 11: 410–418. 22321371 10.1016/j.dnarep.2012.01.008

[75] Dunn OJ (1964) Multiple Comparisons Using Rank Sums. Technometrics 6: 241–252.

