# Supplementary material for: Single Strand Annealing Plays a Major Role in RecA-Independent Recombination between Repeated Sequences in the Radioresistant Deinococcus radiodurans Bacterium
Source: PLoS Genet. 2015 Oct 30;11(10):e1005636. doi: 10.1371/journal.pgen.1005636 (PMC4627823; doi:10.1371/journal.pgen.1005636)

Figure S1

A

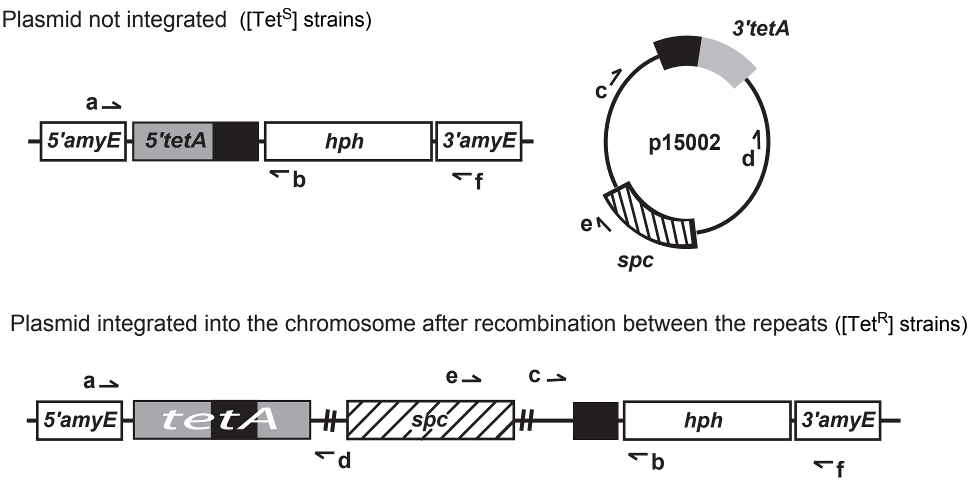

B

	PCR ab	PCR cd	PCR ad	PCR ef
**Plasmid not integrated**	1226 bp	1312 bp	*no PCR product*	*no PCR product*
**Plasmid integrated**	9812 bp	*no PCR product*	1752 bp	3478 bp
**GY15102**	1226 bp	*no PCR product*	*no PCR product*	*no PCR product*
**p15002**	*no PCR product*	1312 bp	*no PCR product*	*no PCR product*

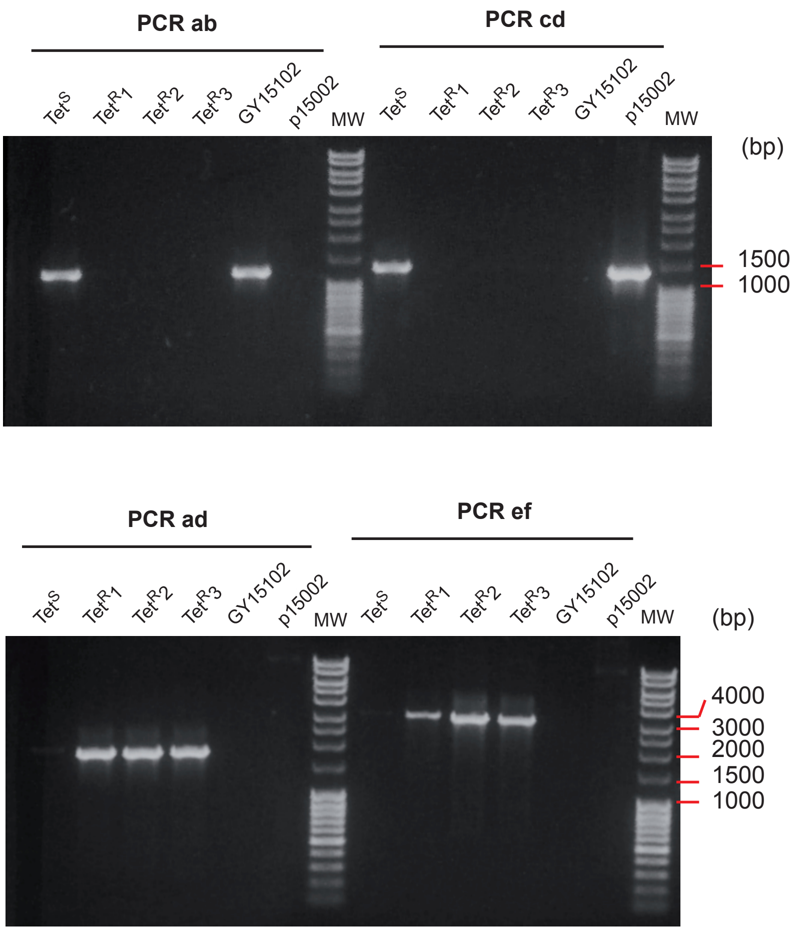

Supplement: S1 Fig — A. Schematic representation of the position of the primer pairs a and b, a and d, c and d, and e and f, used to test the integration of the plasmid in chromosomal DNA B. GY15147 bacteria containing a 5’tetA region inserted in the amyE gene and a 3’tetA region carried by plasmid p15002 were plated on TGY-Agar plates with or without tetracycline. The genomic DNA of 3 independent [TetR] colonies picked and purified on TGY-Agar plates containing 2.5 μg / mL tetracycline and 1 [TetS] colony picked on TGY-Agar plates was purified and used as templates to verify by PCR the integration of the plasmid using the primers pairs described in S1A. Purified p15002 plasmid DNA and genomic DNA of strain GY15102 that does not contain plasmid p15002 were used as controls. PCR fragment sizes are presented for each DNA template. The fragment of 9812 bp was not amplified under the conditions we used for PCR amplification. (PDF) [file pgen.1005636.s001.pdf]
