# Supplementary figures and images for: Single Strand Annealing Plays a Major Role in RecA-Independent Recombination between Repeated Sequences in the Radioresistant Deinococcus radiodurans Bacterium
Source: PLoS Genet. 2015 Oct 30;11(10):e1005636. doi: 10.1371/journal.pgen.1005636 (PMC4627823; doi:10.1371/journal.pgen.1005636)

Figure S2

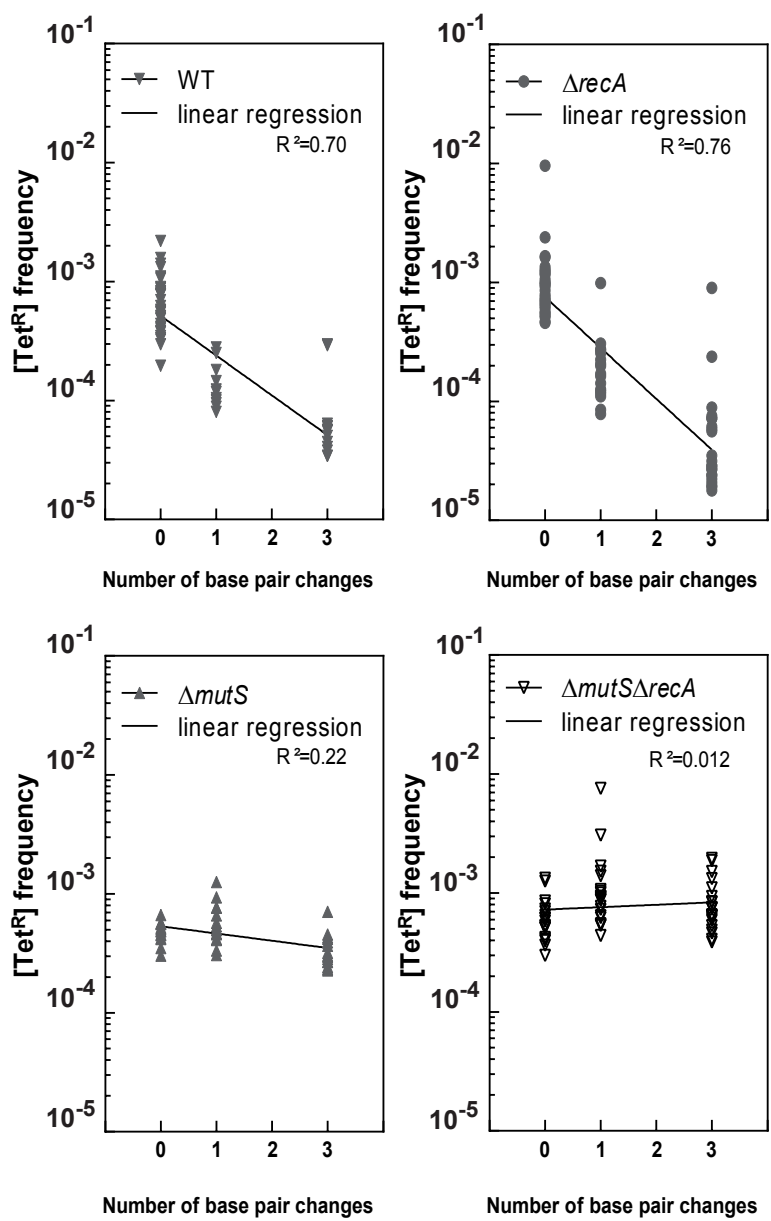

Supplement: S2 Fig — (PDF) [file pgen.1005636.s002.pdf]
