# Supplementary material for: Single Strand Annealing Plays a Major Role in RecA-Independent Recombination between Repeated Sequences in the Radioresistant Deinococcus radiodurans Bacterium
Source: PLoS Genet. 2015 Oct 30;11(10):e1005636. doi: 10.1371/journal.pgen.1005636 (PMC4627823; doi:10.1371/journal.pgen.1005636)

Figure S3

A Diagnostic PCR for double mutants Δ*uvrD*Δ*recA* construction

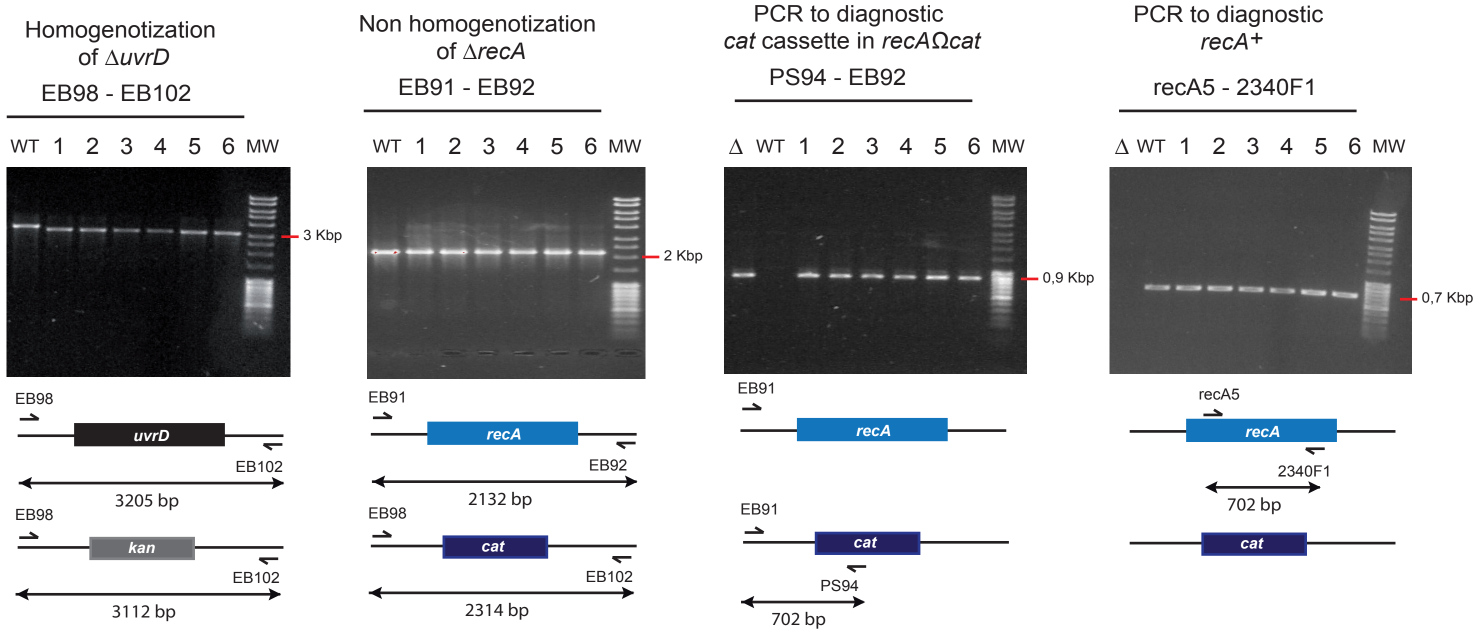

B Diagnostic PCR for double mutants Δ*uvrD*Δ*recF* construction

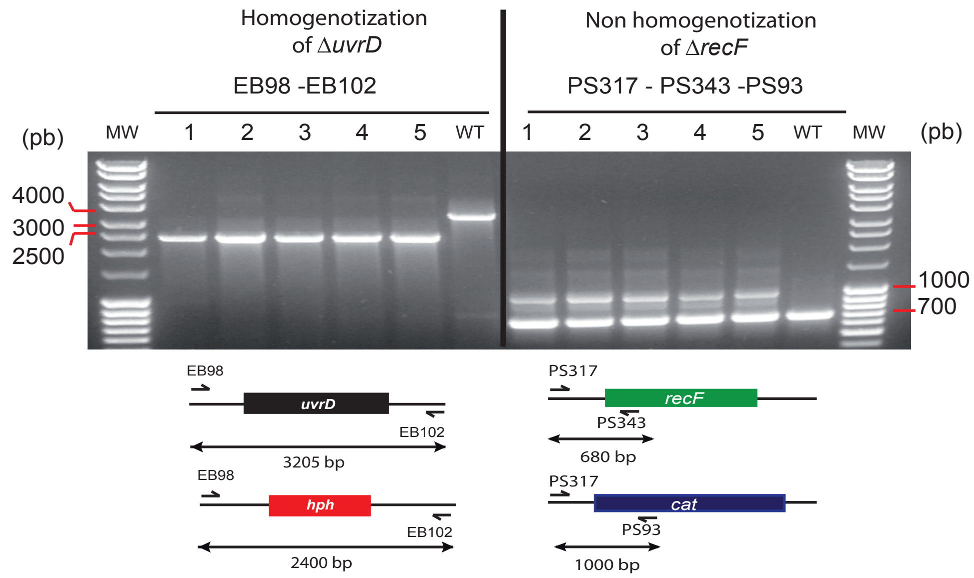

C Diagnostic PCR for double mutants Δ*uvrD*Δ*recR* construction

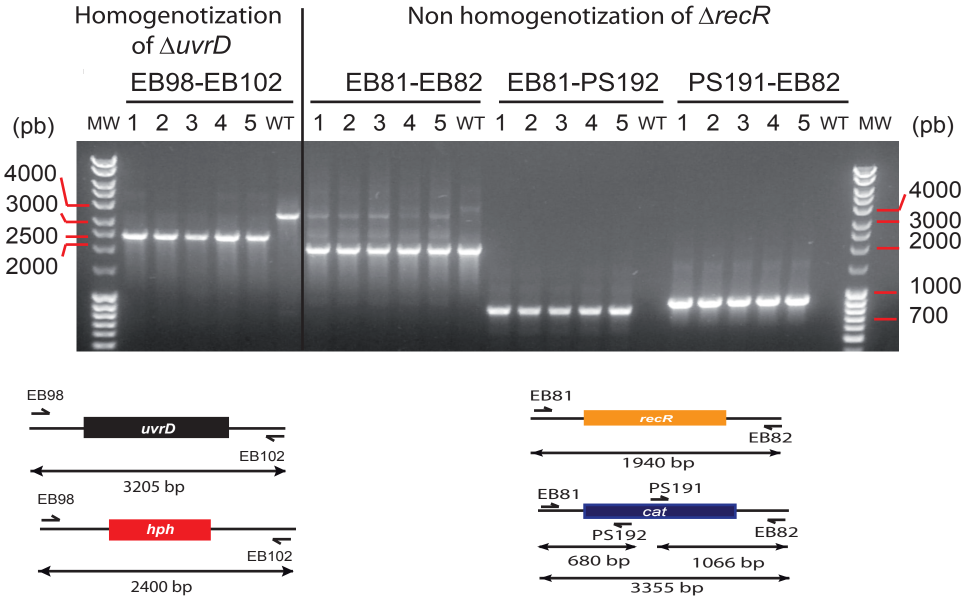

Supplement: S3 Fig — A. Diagnostic PCRs for the deletion of recA and uvrD genes. B. Diagnostic PCRs for the deletion of recF and uvrD genes. C. Diagnostic PCRs for the deletion of recR and uvrD genes. Schematic allelic replacement, primers and PCR fragment sizes are represented for each gene. (PDF) [file pgen.1005636.s003.pdf]
