# Supplementary material for: Single Strand Annealing Plays a Major Role in RecA-Independent Recombination between Repeated Sequences in the Radioresistant Deinococcus radiodurans Bacterium
Source: PLoS Genet. 2015 Oct 30;11(10):e1005636. doi: 10.1371/journal.pgen.1005636 (PMC4627823; doi:10.1371/journal.pgen.1005636)

Figure S4

A Diagnostic PCR for Δ*ddrB*Δ*recA* mutants

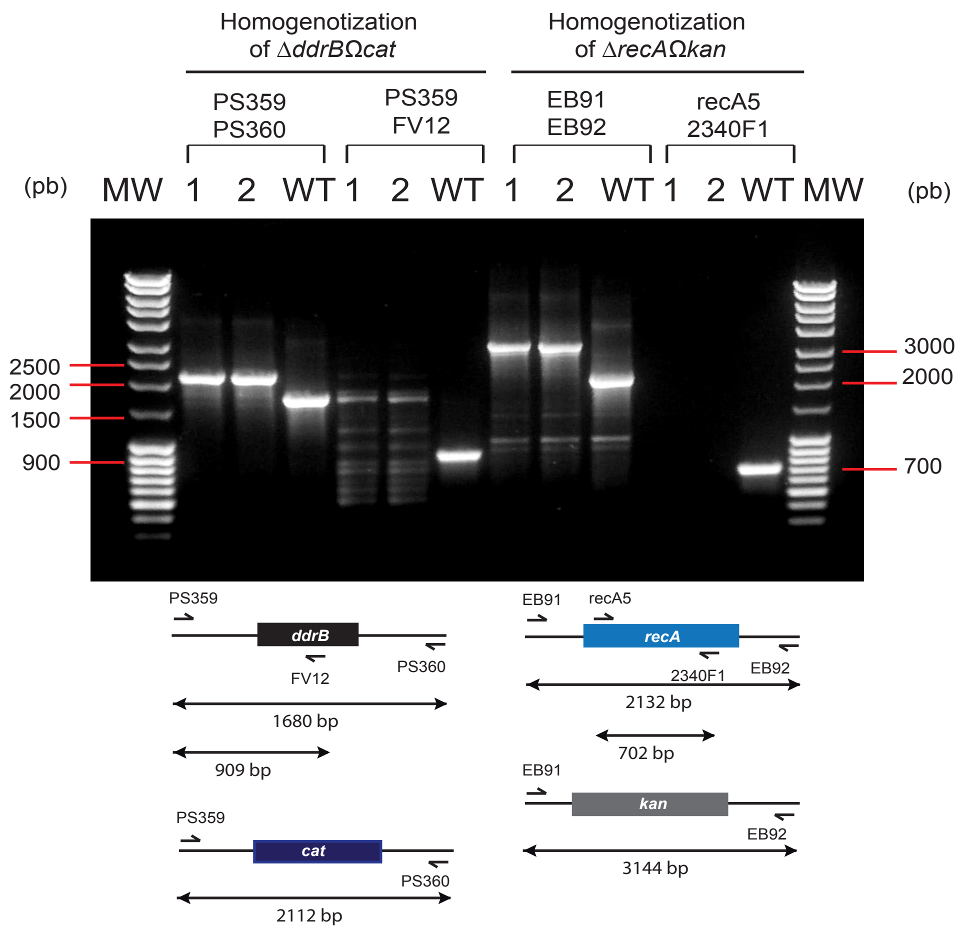

B Diagnostic PCR for Δ*ddrB*Δ*recO* mutants

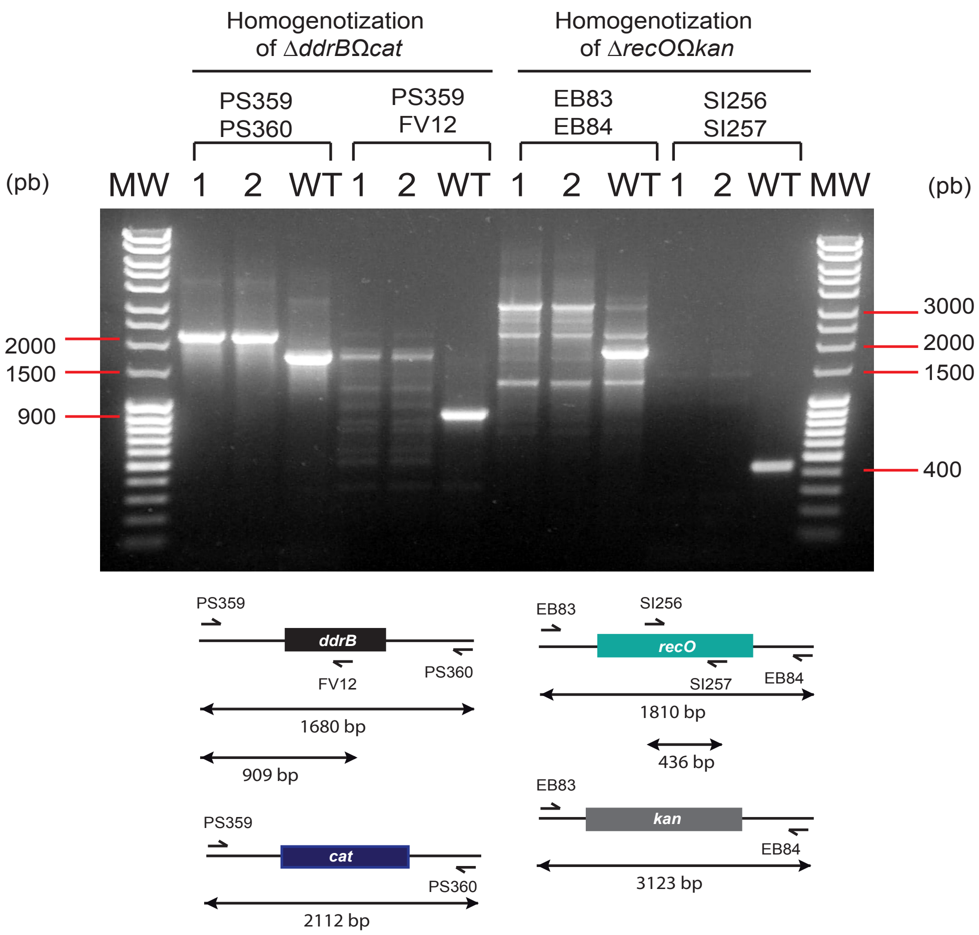

Supplement: S4 Fig — A. Diagnostic PCRs for the deletion of recA and ddrB genes. B. Diagnostic PCRs for the deletion of recO and ddrB genes. Schematic allelic replacement, primers and PCR fragment sizes are represented for each gene. (PDF) [file pgen.1005636.s004.pdf]
